# Genome-wide CRISPR/Cas9 screening for drug resistance in tumors

**DOI:** 10.3389/fphar.2023.1284610

**Published:** 2023-11-21

**Authors:** Zhongyan Zhang, Hailiang Wang, Qian Yan, Jinwei Cui, Yubin Chen, Shiye Ruan, Jiayu Yang, Zelong Wu, Mingqian Han, Shanzhou Huang, Qi Zhou, Chuanzhao Zhang, Baohua Hou

**Affiliations:** ^1^ Department of General Surgery, Guangdong Provincial People’s Hospital, Guangdong Academy of Medical Sciences, Southern Medical University, Guangzhou, China; ^2^ The Second School of Clinical Medicine, Southern Medical University, Guangzhou, China; ^3^ Department of Hepatobiliary Surgery, Weihai Central Hospital Affiliated to Qingdao University, Weihai, China; ^4^ School of Medicine, South China University of Technology, Guangzhou, China; ^5^ Department of Liver Surgery, The First Affiliated Hospital of Sun Yat-Sen University, Guangzhou, China; ^6^ Department of General Surgery, Hui Ya Hospital of the First Affiliated Hospital, Sun Yat-Sen University, Huizhou, Guangdong, China

**Keywords:** drug resistance, tumors, genome-wide CRISPR/Cas9 screening, MAPK pathway inhibitors, PARP inhibitors

## Abstract

Genome-wide clustered regularly interspaced short palindromic repeats (CRISPR)/CRISPR associated nuclease 9 (Cas9) screening is a simple screening method for locating loci under specific conditions, and it has been utilized in tumor drug resistance research for finding potential drug resistance-associated genes. This screening strategy has significant implications for further treatment of malignancies with acquired drug resistance. In recent years, studies involving genome-wide CRISPR/Cas9 screening have gradually increased. Here we review the recent application of genome-wide CRISPR/Cas9 screening for drug resistance, involving mitogen-activated protein kinase (MAPK) pathway inhibitors, poly (ADP-ribose) polymerase inhibitors (PARPi), alkylating agents, mitotic inhibitors, antimetabolites, immune checkpoint inhibitors (ICIs), and cyclin-dependent kinase inhibitors (CDKI). We summarize drug resistance pathways such as the KEAP1/Nrf2 pathway MAPK pathway, and NF-κB pathway. Also, we analyze the limitations and conditions for the application of genome-wide CRISPR/Cas9 screening techniques.

## 1 Introduction

Cancer remains a persistent disease, and treating it will continue to be a formidable challenge. Although molecularly targeted drugs, cellular immunotherapy, and combination approaches have significantly improved cancer prognosis, drug resistance in some tumors still greatly impacts patient outlook.

Drug resistance in tumors is a widespread clinical issue that can be primarily attributed to tumor heterogeneity and the kinetics of cancer cell growth and burden. During progression, tumor cells acquire mutant clones and grow exponentially at low tumor loads. Under drug stress, cells with partially drug-resistant mutations survive and increase exponentially in value. Additionally, new mutations may arise in response to the drug, leading to acquired resistance in the cells (Gottesman, 2002). The typical mechanisms of drug resistance in cancer cells include activation of signaling pathways, loss of function of apoptotic proteins or cancer suppressor genes, the tumor microenvironment and immunology, regulation of microRNAs, secondary mutations affecting drug targets, activation of critical downstream signals, and involvement of histological phenotypes (Ward et al., 2021). It seems that in the presence of drug pressure, genetic mutations occur in tumor cells, which then impact the aforementioned mechanisms to develop drug resistance and promote survival. Given the significance of genetic alterations in cancer drug resistance, gene therapy is a promising new treatment strategy. Compared to conventional treatments such as chemotherapy, gene therapy has fewer adverse effects and offers the potential for a cure (Akbari Kordkheyli et al., 2022). Genetic loss-of-function (LOF) and gain-of-function (GOF) screening is a crucial gene therapy approach that can identify cancer-selective vulnerabilities and holds promise for identifying new therapeutic targets for drug-resistant cancers (Munoz et al., 2016). RNA interference-based screening has been utilized for cancer therapy target identification (Ling et al., 2018), yet its effectiveness is commonly hindered by off-target effects (Munoz et al., 2016). While CRISPR/Cas9 is an efficient and innovative technique for editing genes, it has been utilized to identify mutations responsible for tumor drug resistance (Akbari Kordkheyli et al., 2022). This article reviews the technical characteristics of CRISPR/Cas9, its application in tumor drug resistance, and its limitations. The aim is to offer new ideas for the study of tumor drug resistance using CRISPR/Cas9.

## 2 CRISPR/Cas9 technologies

CRISPR was discovered in prokaryotes and used to counteract exogenous DNA during evolution (Mojica and Montoliu, 2016). The system contains two main components: the single-guide RNA (sgRNA) and Cas9 protein. The 20 bp length sgRNA can recognize the target DNA sequence. The Cas 9 protein, as an endonuclease, can create DNA double-strand breaks (DSBs), which results in non-homologous end-joining or homology-directed repair. Non-homologous end-joining often introduces new bases and leads to insertions and/or deletions (indels) and inactivation of genes, thus enabling targeted gene editing (Hsu et al., 2014). Modifications to the Cas9 protein add more functionality to the CRISPR system. CRISPRa (CRISPR activation) and CRISPRi (CRISPR interference) are two systems based on modified Cas9 protein. The deactivated Cas9 lost its nuclease activity. Instead, it can recruit transcriptional activators or repressors to promote or interfere with gene expression (Konermann et al., 2015).

As the number of sgRNA increases, the scale of libraries increases from small libraries like the kinase library and epigenetic library to genome-wide libraries. Researchers can analyze genetic perturbations on a genome-wide scale in one screening (Han et al., 2016; Yu and Yusa, 2019; Wan et al., 2021).

## 3 The mechanism and strategy of CRISPR/Cas9 screening

**FIGURE 1:**
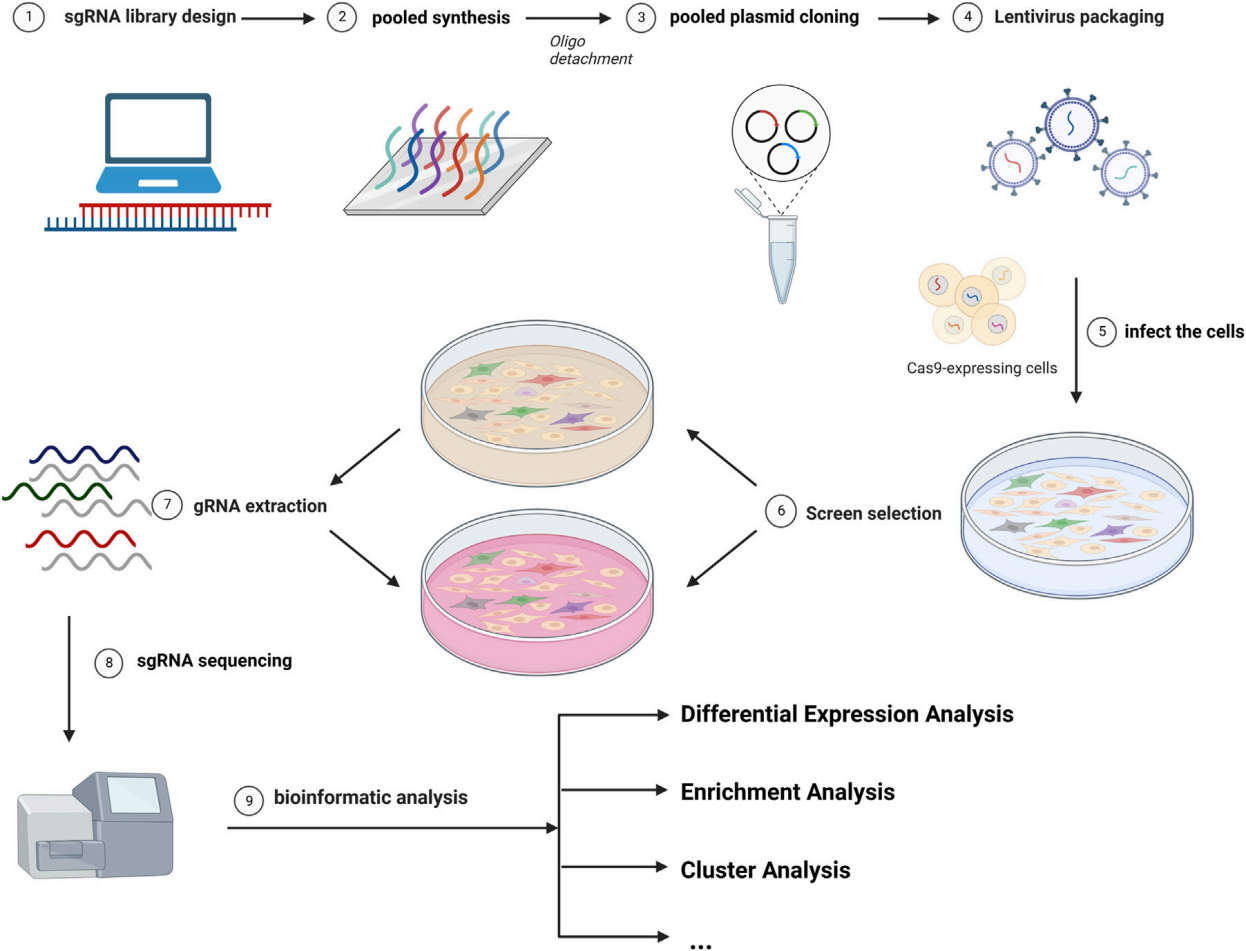
The diagram of common CRISPR/Cas9 screening. The screen workflows include the design of the library, the infection of cells, the selection of cells, the gRNA extraction, and the analysis of the sgRNA distribution.

The most common library is GeCKO (Genome-scale CRISPR Knock-Out) and SAM (synergistic activation mediator), both of which were provided by Zhang (Sanjana et al., 2014; Joung et al., 2017b). Different sgRNAs serve as molecular tags in single cells. The abundance of sgRNAs varies in different subclones in cells. DNA extraction was performed after incubation for different passages and sgRNAs were sequenced. As cells were introduced with different sgRNAs, the knockdown (or overexpression) of drug-promoting and drug-suppressing genes caused changes in drug resistance, leading to differences in cellular growth activity, resulting in different read counts of sgRNA (Li et al., 2014). The read count differences between control and experimental groups were analyzed, and bioinformatic technology and other experiments can be utilized for further inquiry (Shalem et al., 2015). A brief overview of this strategy is depicted in Figure 1.

The screening can be divided into GOF and LOF depending on whether the library is a CRISPRa or a CRISPRi library. In a single screening based on viability, both positive selection and negative selection can be achieved, depending on the increase and decrease in the number of cell populations. The model choice has been well illustrated and the pros and cons of each screening method have been discussed (Doench, 2018; Sharma and Petsalaki, 2018). The model choice depends on the phenotype and perturbation of the system. A pre-well-designed model before screening is of great importance to the next validation of the top-hit genes.

Comprehensively, genome-wide CRISPR/Cas9 knockout (inhibition) or activation screening can locate the gene perturbation caused by drugs. In this review, most studies focus on several notable genes and investigate one gene with drug resistance *in vivo* and *in vitro*.

## 4 Genome-wide CRISPR/Cas9 screening for drug resistance in tumors

In research on drug resistance in tumors, genome-wide CRISPR/Cas9 screening for drug resistance in tumors serves as a powerful initial screening tool for potential drug resistance-related genes. This is of great significance for further treatment of malignant tumors that are resistant to targeted or chemotherapeutic drugs.

We collected data from PubMed by using specific keywords such as CRISPR in combination with other related keywords including pancreatic, lung, liver, gastric, breast, bladder, colon, renal, bone, glioma, ovarian, testicular, cancer, tumor, and malignancy. Only articles focused on drug resistance with genome-wide CRISPR/Cas9 screening were included in this review. After skimming titles and abstracts, 64 articles were included in this review and will be introduced in line with different kinds of drugs, and the summary table is listed in Supplementary Tables S1–S9. We hope that the results will shed light on the resistance mechanism of different drugs.

We focused on the most commonly used drugs in clinical practice and those with the highest number of studies. The drugs involved in this review are MAPK pathway inhibitors, poly (ADP-ribose) polymerase inhibitors (PARPi), antimetabolites, alkylating agents, mitotic inhibitors, immune checkpoint inhibitors (ICIs), and (cyclin-dependent kinase inhibitors) CDKI.

### 4.1 MAPK signaling inhibitors

**FIGURE 2:**
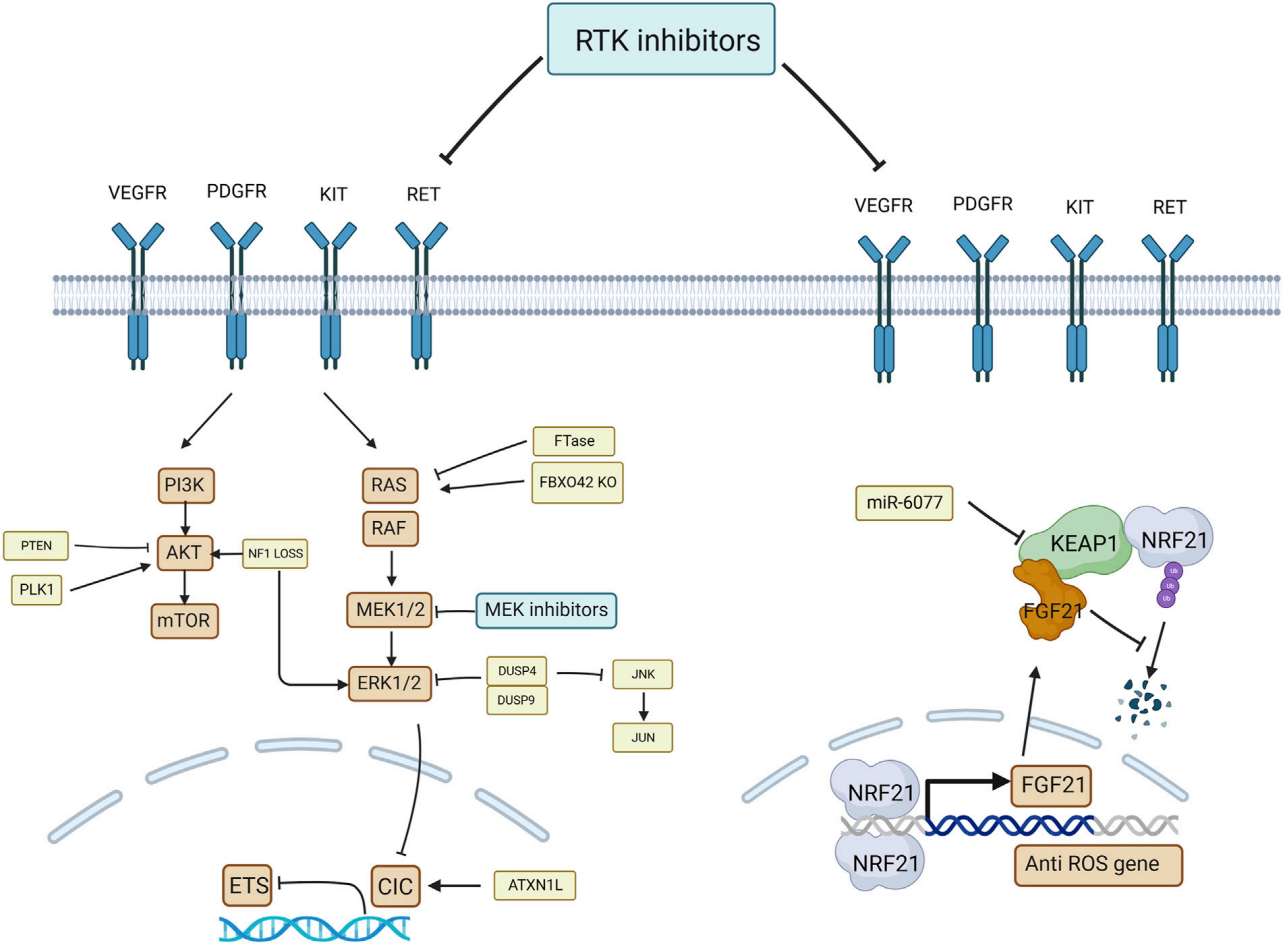
The diagram of the mechanism of MAPK signaling pathway inhibitors. The drug resistance gene involves FBXO42, PLK1, PTEN, DUSP4/9, ATXN1L, and miR-6077. They reverse the drug resistance by reactivating the MAPK pathway or by promoting the transcription of genes that reduce ROS.

MAPK signaling inhibitors are commonly used in clinical practice. Many drugs have been designed to target the RTK (receptor tyrosine kinase)- RAS (rat sarcoma)- RAF (rapidly accelerated fibrosarcoma)-MEK (mitogen-activated protein kinase)-ERK (extracellular-signal-regulated protein kinase) pathway, such as RTK inhibitors, BRAF (B-Raf proto-oncogene) inhibitors, and MEK inhibitors. They act primarily on single or multiple sites such as RTK, Raf1, RafB, and MEK proteins, inhibiting their phosphorylation and blocking signaling. They inhibit angiogenesis, proliferation, and tumor growth (Cargnello and Roux, 2011). Targeted agents are more efficient than traditional chemotherapeutics. However, the clinical efficacy of targeted drugs is unsatisfactory (Zhai and Sun, 2013), and the current genome-wide CRISPR/Cas9 screening model reveals some of the resistance mechanisms. The mechanism of MAPK signaling pathway inhibitors resistance was summarized in Figure 2.

#### 4.1.1 Receptor tyrosine kinase inhibitors

RTK is a class of transmembrane proteins with intrinsic phosphotyrosine kinase activity, including epidermal growth factor receptor (EGFR), vascular endothelial growth factor receptor (VEGFR), fibroblast growth factor receptor (FGFR), MET proto-oncogene (MET), and KIT proto-oncogene (KIT) (Regad, 2015). They mainly receive extracellular signals and regulate cell proliferation, differentiation, and survival. Common RTK inhibitors include erlotinib, lapatinib, and gefitinib. Multi-targeted kinase inhibitors include sorafenib, lenvatinib, and regorafenib.

Erlotinib, the first-generation tyrosine kinase inhibitor (TKI), is mainly used in lung and pancreatic cancer. Terai et al. (2021) identified SHOC2 (leucine-rich repeat scaffold protein) as a drug-resistance gene in lung cancer. SHOC2 affects the sensitivity to EGFR–TKIs in NSCLC (non-small cell lung carcinoma) cells via the SHOC2/MRAS (muscle RAS oncogene)/PP1c (protein phosphatase 1) and SHOC2/SCRIB (scribble planar cell polarity protein) pathways. The mutant SHOC2-MRAS-PP1c complex was later identified to enhance holophosphatase activity (Kwon et al., 2022). Zeng et al. (2019) discovered RIC8A (RIC8 guanine nucleotide exchange factor A) and ARIH2 (Ariadne RBR E3 ubiquitin protein ligase 2) as genes for erlotinib resistance in NSCLC cells. Knockout of RIC8A, essential for G-alpha protein activation, enhanced EGFR TKI-induced cell death, as well as knockout of ARIH2 which conferred resistance to EGFR inhibition by promoting *de novo* protein synthesis through methionyl aminopeptidase 2. By using gefitinib as screening pressure, Georgiou et al. (2020) identified the role of NF1 (neurofibromin 1) in EFGR inhibitor resistance. NF1-mutant colorectal cancer cell lines are resistant to EGFR inhibitors, indicating that loss of NF-1 could be a biomarker for assessing the application of EGFR inhibitors.

Cell cycle proteins and ubiquitination have likewise been found to be associated with erlotinib resistance, which was identified in the screening by Lee J. et al. (2021). Chemical inhibitors targeting genes in these two pathways, nutlin-3 and carfilzomib, in combination with erlotinib reduce the development of erlotinib resistance.

Tumor cells can also acquire a drug-resistant phenotype to RTK inhibitors by EMT (epithelial to mesenchymal transition). Raoof et al. (2019) discovered that FGFR plays a role in tumor mesenchymal cell resistance to a third-generation TKI: EGF816. FGFR signaling was found to be necessary for the survival of epithelial and drug-sensitive cells when undergoing an EMT-like process during the first exposure to EGFR inhibitors, suggesting that dual EGFR + FGFR inhibition may be a promising strategy to prevent the emergence of resistant clones.

#### 4.1.2 Multi-targeted RTK inhibitors

Multi-targeted RTK inhibitors such as sorafenib, lenvatinib, regorafenib, and lapatinib, target more than one kind of RTK, which theoretically inhibit MAPK signaling pathway more effectively, but they also face the dilemma of drug resistance.

A more plausible mechanism is the reactivation of the MAPK/ERK pathway. Lu et al. (2021) screened NF1 and DUSP9 (dual specificity phosphatase 9) by LOF as genes associated with lenvatinib resistance in HCC (hepatocellular carcinoma). Lenvatinib exerts its therapeutic effect mainly by inhibiting kinases in the PI3K (Phosphoinositide 3-Kinase)/AKT (Protein Kinase B) and MEK/ERK signaling pathways. Knockdown of NF1 and DUSP9 increased cell resistance and enhanced cell proliferation and migration. NF1 deletion induced phosphorylation of PI3K/AKT and MAPK/ERK pathways, leading to activation of the pathway and induced lenvatinib resistance. Whereas (Huang et al., 2022) identified DUSP4 (dual specificity phosphatase 4) as an HCC lenvatinib resistance gene through LOF screening, and, similarly, DUSP4 knockdown was found to lead to cellular resistance through activation of the MAPK/ERK pathway. DUSP4 is a member of the bispecific protein phosphatase subfamily involved in the inactivation of the corresponding target kinase, including the MAPK cascade, and inhibition of DUSP4 increased the phosphorylation level of ERK. Lenvatinib blockade of upstream MAPK is not sufficient to inhibit the downstream rephosphorylation of ERK. A combination of lenvatinib and MEK inhibitors is suggested as a possible treatment modality to overcome lenvatinib resistance. A similar mechanism was also found in lapatinib, which is also a multi-targeted RTK inhibitor. Ning et al. (2021) found that loss-of-function mutations in C-terminal Src kinase and PTEN (phosphatase and tensin homolog) in gastric cancer reactivated MAPK and PI3K pathways, leading to lapatinib resistance.

Makhov et al. (2020) found FTase (farnesyltransferase)-dependent cellular factors (a cytokine that acts on RAS proteins) to be associated with sunitinib resistance in clear cell renal cell carcinoma. Combination therapy with lonafarnib, an FTase inhibitor, may potentiate the anti-tumor efficacy of sunitinib through two potential mechanisms: 1) suppression of Rheb-dependent mTOR complex1 activation and 2) dysregulation of lysosomal sequestration of TKIs.

Oxidative stress has been reported to be related to TKI resistance. Many drugs can induce ROS (reactive oxygen species). Zheng et al. (2019) found that KEAP1 (kelch-like ECH-associated protein 1)/Nrf2 (Nuclear factor erythroid 2-related factor 2) affects sorafenib resistance through the ROS pathway. Knockout of KEAP1, a Cul3-based E3 ligase, increased cell survival to sorafenib-resistant treatment by targeting the transcription factor Nrf2 and degradation. Knockdown of KEAP1 led to activation of the cellular Nrf2 pathway that controls the expression of antioxidant genes, resulting in higher resistance to oxidative stress caused by sorafenib. Chen et al. (2022) also identified KEAP1 as a drug resistance-associated gene and FGF21 (fibroblast growth factor 21) as a downstream factor of Nrf2. Upregulation of Nrf2 leads to an increase in FGF21, causing an increase in cellular antioxidant capacity, while FGF21 promotes the transcription of Nrf21 by inhibiting the ubiquitination of Nrf2, leading to high levels of positive Nrf2 feedback, thus promoting a stable state of drug resistance. The KEAP1/Nrf2 pathway is also found in TKI-resistant cells in lung cancer. Krall et al. (2017) investigated the inhibitory effects of EGFR, ALK (anaplastic lymphoma kinase), BRAF, and MEK in lung cancer and found that KEAP1 deletion modulated multiple sensitivities lung cancers with EGFR, ALK, BRAF, and KRAS or NRAS mutations. KEAP1 deletion reduced the ubiquitination of Nrf2 degradation. KEAP1 deficiency promotes cell survival and increases glutathione synthesis thus reducing the drug-induced ROS. In addition, KEAP1 was found to be associated with the miR-6077-mediated pathway for cisplatin/pemetrexed resistance by Bi et al. (2022).

Cellular autophagy, a mechanism of cellular protection in harsh environments, plays a role in TKI resistance in HCC. Li et al. (2021) selected MTX1 (metaxin 1) as a sorafenib resistance-associated gene in HCC through GOF screening. Significant overexpression of MTX1, leading to sorafenib resistance, was associated with poor prognosis and accelerated the proliferation of HCC cells. MTX1 increased drug resistance by antagonizing the inhibitory effect of CDGSH iron sulfur domain 1 on cellular autophagy, which enhances cellular autophagy and increases drug resistance in HCC. The team further identified miR-15a and miR-20b as relevant genes for sorafenib resistance through LOF screening and found that the silencing of miR-15a or miR-20b effectively promoted HCC cell survival and drug resistance in the presence of sorafenib. They both target CDC37L1 (the cell division cycle 37 like 1), which acts as a molecular chaperone in enhancing the interaction between HSP90 and PPIA (peptidylprolyl isomerase A). The silencing of both facilitated the stability of PPIA. Namely, the downregulation of miR-15a and miR-20b promotes the resistance of sorafenib to HCC by enhancing the binding of HSP90 to PPIA via CDC37L1 (Li et al., 2022).

Sun et al. (2018) identified SGOL1 (shugoshin 1), which encodes a mitosis-related protein gene, as a relevant gene for sorafenib resistance in HCC cells by introducing a GECKOv2 sgRNA library into HUH7 cells. SGOL1 knockout can reduce apoptosis and the cytotoxicity of sorafenib. The SGOL1 protein was disabled for the subsequent sister-chromatid segregation at inner centromeres (Zhang and Liu, 2020), suggesting that SGOL1 may modulate drug resistance by regulating chromosome segregation.

Metabolic alternation also plays a role in drug resistance. By performing a genome-wide screening for GECKOv2 in the MHCC97L cell line, Wei et al. (2019) found that PHGDH (phosphoglycerate dehydrogenase), an enzyme of the SSP (serine synthesis pathway), catalyzes the change from 3PG to 3PHP, and knockdown of the PHGDH gene sensitized the HCC cell line to sorafenib. HCC cells respond to oxidative stress induced by sorafenib treatment by increasing PHGDH expression. Activation of SSP is a common mechanism of TKI resistance and targeting SSP through PHGDH inhibitors is one way to treat TKI-resistant HCC. Similarly, Sofer et al. (2022) identified hexokinase 1 and integrin subunit beta 5 as regorafenib resistance-associated genes in a GOF screening. Hexokinase 1 catalyzes the first step of glycolysis, the conversion of glucose to glucose-6-phosphate, suggesting that changes in glucose metabolism underlie drug resistance and that glycolysis inhibitors may improve TKI efficiency.

On the other hand, Cai et al. (2020) used GOF screening to correlate lipid metabolism with cellular resistance and screened LRP8 (LDL receptor-related protein 8) as a sorafenib resistance-associated gene, which encodes a member of the low-density lipoprotein receptor family and functions as a receptor for the cholesterol transporter protein apolipoprotein E. Overexpression of LRP8 inhibited apoptosis and increased sorafenib resistance, whereas knockdown reduced cellular resistance. High levels of LRP8 expression correlated with patient prognosis, and overexpression of LRP8 was found to increase b-catenin levels, and the ApoE-LRP8 pathway was suggested to be a resistance-related pathway in HCC.

#### 4.1.3 RAF inhibitor

RAF inhibitors, such as vemurafenib, mainly inhibit cells with BRAF V600E mutations. In most patients, resistance emerges within a few months. 13 novel genes were identified by Goh et al. (2021) in screening for vemurafenib resistance in melanoma cells. Among them, NF1, CUL3, and NF2 are associated with the MAPK pathway, and the remaining genes are related to epigenetics, cell cycle, telomeres, etc. Joung et al. (2017a) identified 11 LncRNA loci in melanoma cells. Gautron et al. (2021) identified SMAD3, BIRC3, and SLC9A5 in melanoma as key actors of BRAF inhibitor resistance. SMAD3 plays a key role in melanoma resistance to treatment by promoting an EMT-like process, and their results suggest that the regulation of BRAF inhibitor resistance gene expression is multiparametric.

#### 4.1.4 MEK1/2 protein inhibitors

Trametinib, a MEK inhibitor, functions as an allosteric, ATP noncompetitive inhibitor with nanomolar activity against both MEK1 and MEK2 kinases. It can inhibit cell proliferation, cause cell cycle arrest in the G1 phase, and induce apoptosis. Wang et al. (2017) performed a genome-wide screening in KRAS-mutated pancreatic adenocarcinoma to investigate RTK-RAS-MAPK pathway reactivation and found that gene ATXN1L deletion caused a decrease in protein CIC (capicua transcriptional repressor), leading to increased cellular resistance, and determined the ATXN1L-CIC-ETS transcription factor axis to be a mediator of resistance to MAPK inhibitors. The ERN1-JNK-JUN pathway is present in KRAS mutant colorectal cancers and is involved in regulating MEK inhibitor resistance in colon cancer (Šuštić et al., 2018). It is emphasized that JUN-activated kinases, TAK1 and JNK, may be important loci for MEK inhibitors in KRAS-mutant cancer cells.

FBXO42 (F-box protein 42) is an E3 ubiquitin ligase associated with trametinib resistance in NRAS-mutated melanoma cells. Knockdown of FBXO42 increases the TAK1 signaling pathway, which may promote an increase in active P38, leading to an enhancement of the ERK signaling pathway. This suggests that the concomitant use of MEK inhibitors with TAK1 inhibitors can improve the efficacy of MEK inhibitors (Nagler et al., 2020). Yu et al. (2022) found that GRB7 (growth factor receptor bound protein 7) effectively increased major resistance to MEK inhibitors via the RTK pathway in KRAS-mutated colon cancer. PLK1 (Polo-like kinase 1) is the major interacting kinase of GRB7. The combination of PLK1 and MEK inhibitors could synergistically inhibit CRC cell proliferation and induce apoptosis *in vitro* and *in vivo*.

### 4.2 Poly (ADP-ribose) polymerase inhibitors

**FIGURE 3:**
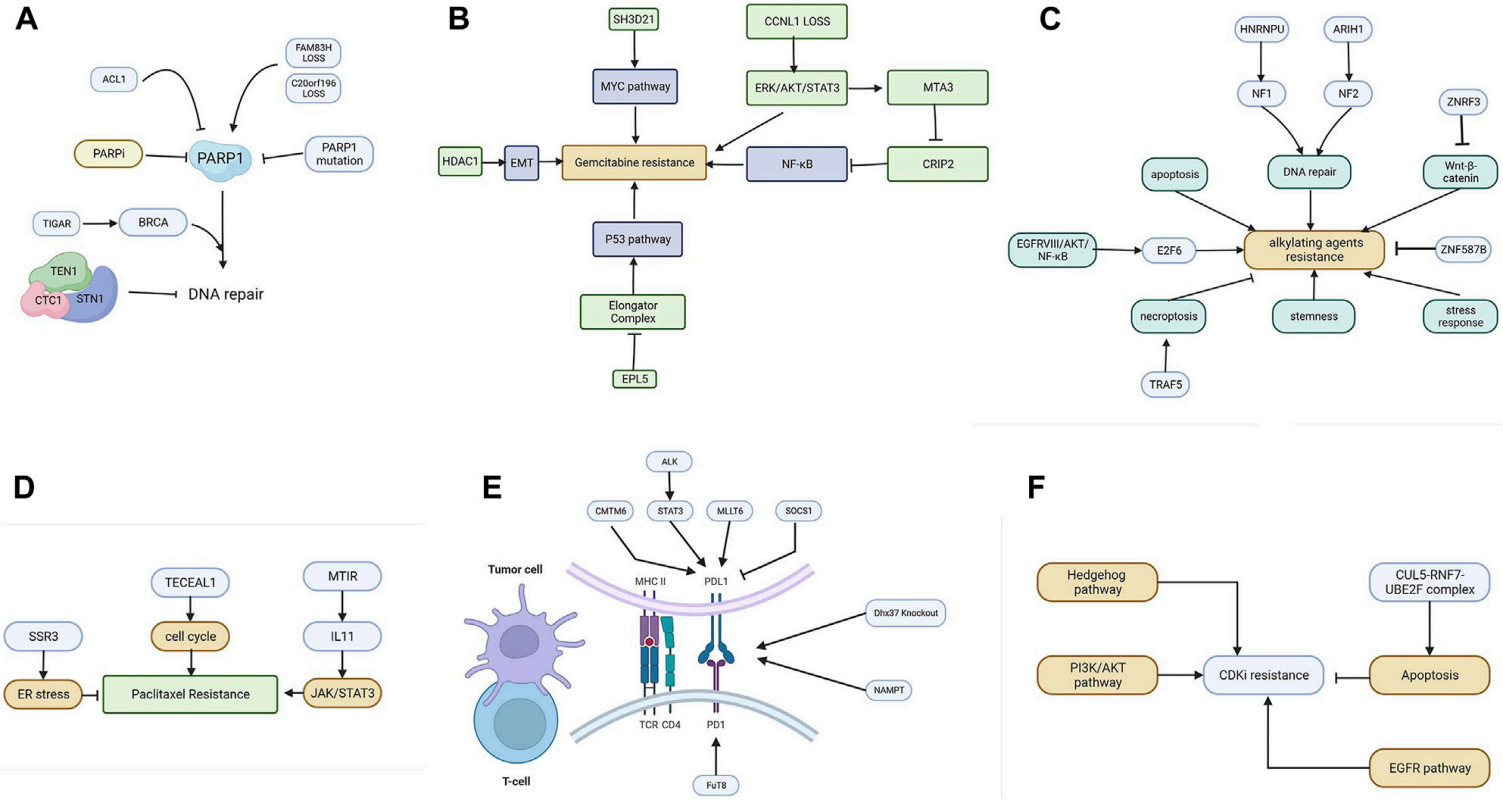
**(A)** The resistance mechanism of PARP inhibitors. **(B)** Various genes promote gemcitabine resistance by activating the EMT, NF-κB, MYC, and P53 pathways. **(C)** The resistance mechanism of alkylating agents includes the enhanced pathway of DNA repair, apoptosis, Wnt-β catenin, NF-κB, stemness, and stress response. **(D)** Cell cycle, JAK/STAT3, and ER stress are involved in mitotic inhibitor resistance. **(E)** The resistance mechanism of ICIs. Tumor cells can upregulate PD-L1 to inhibit T-cells via CMTM6, ALK, MLLT6, and SOCS1. **(F)** The resistance mechanism of CDKI. The Hedgehog pathway, PI3K/AKT pathway, apoptosis, and EGFR pathway are involved in CDKI resistance.

The mechanism of PARP inhibitors is related to the concept of synthetic lethality. Protein PARP1 (poly (ADP-ribose) polymerase 1) is mainly responsible for the repair of DSBs. PARPi targeting homologous recombination-deficient tumors hold great promise for the treatment of tumors with damaging mutations in BRCA1/2 or other homologous recombination factors (Noordermeer and van Attikum, 2019). Unfortunately, PARPi resistance has proven to be a major clinical problem. The use of genome-wide CRISPR/Cas9 screening techniques helps us to better understand the cell response to PARPi. The mechanism of PARPi is summarized in Figure 3A.

The resistance mechanism of PARPi mainly focuses on the promotion of DNA repair. Barazas et al. (2018) performed LOF screening in a BRCA1-deficient cell line and found that the deletion of members of the CTC1-STN1-TEN1 complex led to PARPi resistance in BRCA1-deficient cells *in vitro* and *in vivo* by enhancing the repair of DSBs. Dev et al. (2018) identified two proteins, C20orf196 and FAM35A, in BRCA1-deficient cells by performing a whole genome screening in breast cancer. The inactivation of the two proteins resulted in strong PARP inhibitor resistance by forming a complex, “Shieldin” (SHLD1/2), which promotes DSBs end-joining.

Mutation of the PARP1 protein that results in drug resistance has been illustrated. By picking resistant clones in genome-wide screening in embryonic cells, Pettitt et al. (2018) found PARP1 point mutations leading to PARP inhibitor resistance in ovarian cancer. Mutations both within and outside of the PARP1 DNA-binding zinc-finger domains cause PARPi resistance and alter PARP1 trapping, suggesting that PARP1 intramolecular interactions may influence PARP-mediated cytotoxicity.

Zimmermann et al. (2018) performed three screenings with the TKOv1 library and discovered that mutations in three genes encoding ribonuclease H2 sensitized cells to PARP inhibition and the manipulation of genomic ribonucleotide processing may contribute to the treatment with PARPi.

Fang et al. (2019) found that TIGAR (TP53-induced glycolysis regulatory phosphatase), encoded by C12orf5, regulates PARP1 resistance in ovarian cancer, and high expression of TIGAR is associated with poor prognosis. TIGAR knockdown enhanced the sensitivity of cancer cells to olaparib through the downregulation of BRCA1 and Fanconi anemia depletion pathways and increased the sensitization of these cells by affecting metabolic pathways and increasing the cytotoxic effects of olaparib.

Juhász et al. (2020) identified ALC1 (amplification in liver cancer1) as a modulator of PARP inhibitor. ALC1 can remove inactive PARP1 through binding to PARylated chromatin, thus the overexpression of ALC1 reduces the trapping of inhibited PARP1 and decreases the sensitivity of BRCA-deficient cells to PARP inhibitors.

Autophagy plays an important role in treatment with olaparib and is associated with its resistance. In prostate cancer, Ipsen et al. (2022) determined that deletion of PARP1, ARH3 (ADP-ribosylserine hydrolase), tryptophan 5-monooxygenase activation protein epsilon, and ubiquitin protein ligase E3 component n-recognin 5 resulted in olaparib resistance, where PARP1 or ARH3 knockdown resulted in reduced autophagy and increased cellular resistance, suggesting that low ARH3 expression is an independent prognostic indicator.

### 4.3 Antimetabolites

Gemcitabine, 2′,2′-difluoro-2′-deoxycytidine, is currently used in a variety of solid tumor cancers. It is delivered into cells via membrane nucleoside transport proteins (hENTs and hCNTs). It undergoes complex conversion to the nucleotides gemcitabine diphosphate and triphosphate (Mini et al., 2006). The triphosphate competes with deoxycytidine triphosphate, which leads to inhibitory DNA synthesis. Several screenings have been performed to elucidate the mechanism of gemcitabine resistance. The mechanisms are summarized in Figure 3B.

Bakke et al. (2019) identified PSMA6 (proteasome 20S subunit alpha 6) in pancreatic cell lines and validated its role in pancreatic cancer cell lines and found that the knockout of PSMA6, as a proteasomal subunit of the 20S core complex, can induce apoptosis in cells.

SH3D21 (SH3 domain containing 21) (Masoudi et al., 2019) was identified as a drug resistance maintenance gene in the Panc1 cell line, and knockdown of SH3D21 resulted in increased sensitivity of pancreatic cancer cells to gemcitabine, and it is hypothesized that the MYC pathway is associated with gemcitabine resistance. Yang et al. (2022) screened for DCK (deoxycytidine kinase) and cyclin L1 and found that deletion of CCNL1 activated the ERK/AKT/STAT3 survival pathway, leading to cellular resistance to gemcitabine treatment.

The MTA3 (the metastasis associated 1 family member 3)-CRIP2 (cysteine-rich protein 2)-NF-κB pathway was associated with gemcitabine resistance. Wu et al. (2022) screened MTA3 through genome-wide overexpression as part of the NuRD transcriptional repressor complex. Downregulation of the MTA3 gene resulted in increased cellular sensitivity to gemcitabine. It was further determined that MTA3 primarily represses CRIP2 transcription whereas CRIP2, a transcriptional repressor, primarily suppresses tumorigenesis by inhibiting NF-κB signaling to suppress tumorigenesis. Sarr et al. (2019) found a role for pyrimidine metabolism in NUC-1031 resistance, which is a phosphoramidite transformation of gemcitabine, mainly through the DCK and dCTP pyrophosphatase 1, and concluded that DCK levels were associated with patient prognosis.

Xu et al. (2019) found that the elongator complex was associated with gemcitabine resistance. They identified ELP5 (elongator acetyltransferase complex subunit 5) in gallbladder cancer. The loss of ELP5 compromised the integrity and stability of the elongator complex and abrogated wobble U34 tRNA modification, which interfered with the translation of hnRNPQ (heterogeneous nuclear ribonucleoprotein Q) mRNA, which in return regulates cells through the P53 pathway. The elongator/hnRNPQ/P53 axis may control gemcitabine sensitivity.

Ramaker et al. (2021) performed CRISPR screening among four classes of chemotherapeutic agents (gemcitabine, oxaliplatin, irinotecan, and 5-fluorouracil), and found that HDAC1 (histone deacetylase 1) and ABCG2 (ATP binding cassette subfamily G member 2) serve as four common drug resistance genes, with HDAC1 overexpression leading to drug resistance associated with EMT, while ABCG2 was found to be a general resistance mechanism mainly through the cells that pump the drug.

### 4.4 Alkylating agents

Common alkylating agents include cisplatin, oxaliplatin (a third-generation platinum-based chemotherapeutic agent), and temozolomide. The pharmacological mechanism of alkylating agents involves the inhibition of DNA replication and transcription through internal and inter-strand crosslinks resulting from binding to DNA (Wang L. et al., 2021), followed by the induction of damage to double-stranded DNA. Cisplatin is a first-generation platinum drug used as a first-line therapy in clinical practice with a good inhibitory effect on solid tumors. Figure 3C summarizes the resistance mechanism of common alkylating agents.

Non-coding RNAs also play a role in regulating drug-resistant cisplatin resistance. Bi et al. (2022) found that miR-6077 promoted cisplatin/pemetrexed resistance in lung adenocarcinoma through the cyclin-dependent kinase inhibitor 1A and KEAP1 pathways. Goodspeed et al. (2019) found MSH2 (mutS homolog 2) in bladder cancer that led to *in vitro* resistance to cisplatin via the hyaluronan-mediated motility receptor pathway and that patients with low MSH2 levels had a poor prognosis when receiving platinum-based chemotherapy. Also in bladder cancer, Shi et al. (2022) found that HNRNPU (heterogeneous nuclear ribonucleoprotein U) knockdown inhibited cell proliferation, invasion, and migration. Furthermore, the deletion of HNRNPU promoted the sensitivity of T24 cells to cisplatin, mainly associated with S cell cycle phase blockage, and in addition, HNRNPU was found to regulate chemosensitivity by affecting the expression of NF1.

In melanoma, zinc and ring finger 3, an ubiquitin ligase known to be a targeting and negative feedback regulator of Wnt-β catenin signaling enhanced cisplatin resistance in normal and melanoma cells independently of b-catenin. ARIH1 (Ariadne1 homolog), another ubiquitin ligase, also enhanced cisplatin resistance in normal and melanoma cells by modulating ARIH1, and the tumor suppressor neurofibrillary protein 2, NF2, enhanced cisplatin resistance in melanoma, but not in normal cells (Ko and Li, 2019).

Lan et al. (2021) identified M2 tumor-associated macrophages as an important mediator of oxaliplatin resistance acquisition in colorectal cancer. Moreover, TNF receptor-associated factor 5 mediates oxaliplatin resistance in CRC triggered by methyltransferase 3.

In ovarian cancer, Ouyang et al. (2019) found that downregulation of SULF1 (sulfatase 1) resulted in diminished cisplatin-induced cytotoxicity. SULF1 may regulate cell signaling by altering the sulfated state of the acetyl heparan sulfate chain, thereby affecting platinum sensitivity; in addition, knockdown of ZNF587B, a C2H2-type zinc finger protein, significantly reduced cisplatin sensitivity in ovarian cancer cells. Also in ovarian cancer, Stover et al. (2019) performed systematic overexpression and inhibition of BCL-XL, BCL-W, MCL1 (myeloid cell leukemia sequence 1), or BCL-2 and found that overexpression of anti-apoptotic proteins increased cisplatin and paclitaxel resistance.

Skripova et al. (2022) performed a screening for cisplatin and oxaliplatin resistance in pancreatic cancer. The genes were associated with DNA repair, cell cycle regulation, components of detoxification and antioxidant systems, and intracellular signaling pathways. The results also identified genes previously associated with platinum drug sensitivity/resistance, demonstrating the adequacy of the CRISPR/Cas9 screening approach in the search for regulators of drug sensitivity.

Temozolomide (Lee, 2016) (TMZ) is an oral alkylating agent used to treat glioblastoma multiforme and astrocytoma. However, at least 50% of TMZ-treated patients do not respond to TMZ. This is mainly due to the overexpression of O-6-methylguanine-DNA methyltransferase and the lack of DNA repair pathways in glioblastoma cells.

Huang et al. (2019) identified in glioblastoma that NF-κB/E2F6 (E2F transcription factor 6) was responsible for EGFRvIII-associated temozolomide resistance and E2F6, under the control of the EGFRvIII/AKT/NF-κB pathway, showed a promising therapeutic target for TMZ resistance.

MacLeod et al. (2019) described genome-wide CRISPR/Cas9 screening, identifying genetic vulnerabilities in a panel of patient-derived glioblastoma stem cell cultures. Regulators of stemness (genes such as SOX2, SOX9, DOT1L, and SOCS3) and stress response (UFMylation and endoplasmic reticulum-associated protein degradation pathway) govern the growth of glioblastoma stem cells. Chemogenomic screening using temozolomide identified modulators of sensitivity to chemotherapy. In the over-expression group, the Nrf2 and Wnt pathways were involved in TMZ resistance, and overexpression of frizzled class receptor 6, catenin beta 1, or Nrf2 genes significantly increased cell survival (Rocha et al., 2020).

### 4.5 Mitotic inhibitors

One of the most well-known mitotic inhibitors is paclitaxel (PTX). PTX (Abu Samaan et al., 2019) is widely used in the treatment of various types of malignant diseases. The mechanism of PTX action represents several ways in which PTX affects cellular processes leading to programmed cell death. PTX is frequently used as a front-line therapeutic agent in breast cancer. Unfortunately, the resistance of BC to PTX therapy is a major barrier to clinical use and one of the leading causes of death associated with treatment failure. Factors that contribute to PTX resistance are ABC transporter proteins, microRNAs, or mutations in certain genes. The resistance patterns are summarized in Figure 3D.

Rushworth et al. (2020) identified 17 candidate genes in prostate cancer whose inhibition may enhance the efficacy of docetaxel, with TCEAL1 (transcription elongation factor A-like 1) being the preferred candidate. Deletion of TCEAL1 leads to altered cell cycle, increased sub-G1 cell death, and increased polyploidy.

Paclitaxel resistance is a major concern in the treatment of patients with breast cancer, and (Lian et al., 2020) showed that expression of MEF2-interacting transcriptional repressor can increase the level of interleukin 11 and activate the downstream JAK/STAT3 signaling in triple-negative breast cancer, which can lead to paclitaxel resistance.

Dmello et al. (2022) found that the endoplasmic reticulum protein SSR3 (signal sequence receptor subunit 3) was associated with paclitaxel resistance in breast cancer, and that knockdown of SSR3 made cells resistant to PTX, while overexpression made them sensitive to PTX. The mechanism is that SSR3 confers susceptibility to PTX by regulating the phosphorylation of ER stress sensor IRE1α.

### 4.6 Immune checkpoint inhibitors

Tumor cells escape from immune surveillance and progress through different mechanisms, including activation of immune checkpoint pathways that suppress antitumor immune responses. ICIs enact anti-tumor functions by interrupting co-inhibitory signaling pathways and promoting immune-mediated elimination of tumor cells. Figure 3E summarizes the network of resistance in tumor and immune cells.

Immune checkpoint inhibitors target different axes including cytotoxic T lymphocyte antigen 4, PD-1/PD-L1 (programmed death protein 1/programmed death ligand 1), B-and T-lymphocyte attenuator, T cell immunoglobulin and mucin-containing molecule 3, T cell immunoreceptor with Ig and ITIM domains, V-domain Ig suppressor of T-cell activation, lymphocyte activation gene-3, and indoleamine 2,3-dioxygenase 1 (Lee J. B. et al., 2021). While resistance frequently occurs in patients treated with conventional cancer therapies and targeted therapies, in large subsets of patients treated with ICIs, long-lasting immunologic memory is commonly identified (Jenkins et al., 2018). However, the emergence of acquired resistance is observed in longer follow-up clinical trial populations.

Genome-wide CRISPR screening for ICIs can be performed both in immune cells and tumor cells to locate related axes that regulate acquired resistance.

Deficiencies in cancer cell antigen presentation are the main mechanism of ICI resistance. Dong et al. (2019) performed *in vivo* and *in vitro* CRISPR screening in CD8 T cells and found that knockdown of DEAH-box helicase 37 enhanced the efficacy of antigen-specific CD8 T cells against triple-negative breast cancer *in vivo*. Wang Y. et al. (2021) determined that nicotinamide phosphotransferase is required for T-cell activation and demonstrated that NAD + supplementary significantly enhanced anti-PD-1 immunotherapy in a murine solid tumor model. Okada et al. (2017) found that the inhibition of fucosyltransferase 8, by genetic ablation or pharmacologic inhibition, could reduce cell-surface expression of PD-1 and enhance T-cell activation, leading to more efficient tumor eradication.

A screening in melanoma cells by Manguso et al. (2017) found that the deletion of protein tyrosine phosphatase protein tyrosine phosphatase non-receptor type 2 (PTPN2) enhances interferon-γ-mediated antigen presentation and growth inhibition to improve the efficacy of immunotherapy. Burr et al. (2017) found in a genome-wide screening that CMTM6 (CKLF-like MARVEL transmembrane domain containing 6 protein) could bind PD-L1 and maintain its expression on the cell membrane surface, and that the reduction of CMTM6 reduced PD-L1 expression and attenuated tumor suppression of T cells. Gu et al. (2021) found that TNF receptor-associated factor 3, with its regulation of the NF-κB pathway, led to a decrease in the MHC-I-specific negative regulator TRAF, which sensitized cancer cells to antigen-specific T cell-driven cytotoxicity. This finding may be useful in the treatment of MHC-I-suppressed tumors. Using genome-wide CRISPR and metabolic inhibitor screening, Zhang et al. (2019) demonstrated that ALK activates STAT3 and ultimately induces PD-L1 expression through the effect of interferon regulatory factor 4 and basic leucine zipper ATF-like transcription factor 3 on the enhancer region of the PD-L1 gene. Suresh et al. (2020) identified that impairment of heme production activated an integrated stress response that allowed bypassing of the inhibitory upstream open reading frame in the PD-L1 5' UTR, thereby enhancing PD-L1 translation and suppressing anti-tumor immunity. Wroblewska et al. (2018) developed a barcoding system that operates at the protein level and identified SOCS1 (suppressor of cytokine signaling 1) as a negative regulator of PD-L1. Frequent loss of interferon regulatory factor 2, resulting in reduced MHC I antigen presentation and increased PD-L1 expression leading to immune escape (Kriegsman et al., 2019). Sreevalsan et al. (2020) found that the transcriptional regulator MLLT6 (myeloid/lymphoid or mixed-lineage leukemia; translocated to, 6) is required for efficient PD-L1 protein expression and cell surface presentation in cancer cells. Depletion of MLLT6 reduced the inhibition of CD8 cytotoxic T cell-mediated cytolysis. Taken together, the above shows that CRISPR not only has a screening function in the drug-tumor cell model but also helps to screen immune cells and tumor cells for regulatory factors in immunotherapy.

### 4.7 CDK inhibitors

Cell cycle regulation depends on three major regulators, namely, cyclin, CDK (cell cycle-dependent protein kinases), and CDKI, and the normal function of the cell cycle depends mainly on the temporal activation of various CDKs and their phosphorylation modifying the corresponding substrate protein kinase complexes of cyclins and CDK. The mechanism of CDKI is shown in Figure 3F.

The first generation of CDKIs, such as flavopiridol and roscovitine, demonstrated disappointing effects in clinical trials due to their defects in targeting specific CDKs, while the third generation of CDK4/6 inhibitors, such as palbociclib, abemaciclib, and ribociclib, had satisfactory results in advanced or metastatic breast cancer.

Tong et al. (2019) found differential genes in bladder cancer were mainly enriched in the receptor tyrosine kinase, PI3K-Akt, Ras/MAPK, JAK/STAT, or Wnt signaling pathways and found that inhibitors targeting RTKs, PI3K-AKT and Ras/MAPK had synergistic effects in combination with palbociclib. Kabir et al. (2019) found that the cullin 5 ubiquitin ligase complex mediated the resistance of lung cancer cells to cyclin-dependent kinase 9 and MCL1. Daggubati et al. (2021) performed a CDK4/6 inhibitor screening in medulloblastoma and identified reduced ribosomal protein expression as the basis of resistance to CDK6 inhibition in Hedgehog-associated medulloblastoma cells, leading to ER stress and activation of the unfolded protein response. This increases the activity of enzymes that produce the smooth-activated sterol lipids which maintain HH signaling in medulloblastoma. Upregulation of RTK-RAS-RAF and RTK-PI3K-AKT signaling cascade activity in NRAS mutant melanoma leads to resistance to combined inhibition of MEK1/2 and CDK4/6 (Hayes et al., 2019). Mayayo-Peralta et al. (2021) identified activation of the EGFR pathway in CDK4/6-resistant cells in breast cancer, raising the possibility of using receptor tyrosine kinase signaling cascade inhibitors to target CDK4/6 inhibitor resistance.

## 5 Discussion

The above results demonstrate the power and convenience of genome-wide CRISPR/Cas9 screening as a screening technique. Based on our data, numerous drug-resistance genes have been identified in multiple tumors based on CRISPR/Cas9 screening technology, which affects the cellular response to chemotherapeutic agents through various pathways. The most abundantly studied are MAPK pathway inhibitors, and the common mechanisms of resistance are the reactivation of MAPK pathways such as DUSP7, NF2, and the KEAP1/Nrf2 pathway, which are present in multiple cancer resistance models. Despite the variation in results between studies, individual genes have been validated *in vitro* and *in vivo* and do correlate with cellular drug resistance. This suggests that the acquisition of cellular drug resistance phenotypes is often a multi-pathway, multi-omics alteration, such as metabolism, EMT, and signal transduction.

However, CRISPR/Cas9 screening still has some shortcomings that need to be addressed. The genome-wide screening technique is highly influenced by cell heterogeneity, resulting in different sgRNA distributions even within identical studies, cell lines, and drug treatments (Sun et al., 2018; Zheng et al., 2019). The screening results also depend on several factors such as the construction of the sgRNA library, the treatment of the control group, the cell response to the delivery system, the multiplicity of infection, and the random genetic drift of the cell line (Doench, 2018). The most important of these is the establishment of the sgRNA library since the downstream data analysis mainly focuses on the distribution of the sgRNA in the genome by sequencing. The off-target effect, the efficiency of the knockout effect, the drug concentration, and the time length for drug exposure should also be taken into consideration.

A model of cellular alteration to drug resistance suggests that cells acquire drug resistance partially through alterations in the epigenome in the early stages and phenotypic alterations in drug resistance through alterations in the genome, transcriptome, and metabolome in the later stages (Wang et al., 2023). To better illustrate this genetic alternation pattern, a more comprehensive approach can be done by single-cell screening. Single-cell CRISPR/Cas9 screening enables the detection of both the sgRNA distribution and the transcriptome data and is an exciting technology but also has a high sequencing cost (Wang and Wang, 2017). Another screening method is arrayed screening. While all sgRNAs are introduced into a single culture dish in a pooled screening, array screening introduces each sgRNA into cells in a single well on a platform. This technology allows for the selection of phenotypes not limited to cell viability, such as subcellular localization and morphometric (Datlinger et al., 2017). The application for different screening methods depends on the selection criteria. Pooled screening, due to its lower cost and ease of operation, has become the most used method for drug resistance research.

Also, targeting partial genomic screening has higher robustness than whole-genome screening (Zhong et al., 2022), and whole-genome screening tends to cover most of the differential genes, but this methodology may not uncover very strongly selected genes and may be dependent on factors such as *in vitro* drug metabolism effects (Sarr et al., 2019). For genome-wide screening assays, targeted experiments on the screened genes are required to draw more rigorous conclusions.

Therefore, treatments targeting a single gene (e.g., single-point targeted drugs) may eventually lead to the emergence of new drug-resistant genes, and the change in tumor drug resistance can only be to a certain extent and cannot fundamentally change the cellular drug resistance. If we want to reverse the process of tumor drug resistance, it is essentially a process of reversing entropy, which requires precise modulation of the dysregulated gene within the tumor to reprogram the expression network, which is currently pending the emergence of new technologies.

Genome-wide CRISPR/Cas9 screening technology is a powerful tool for screening unknown drug resistance genes and has particularly progressed in different cancer studies, especially in targeted drug resistance studies. Drug resistance in tumor cells is often caused by multiple gene mutations. Meta-analysis against genome-wide CRISPR/Cas9 drug resistance screening models can perhaps construct a gene-related network to reveal the drug resistance network in tumors.

CRISPR/Cas9 technology currently has powerful capabilities as a gene editing technology, and genome-wide screening technology is also a credible and convenient means of screening cellular drug resistance-associated genes. More data from CRISPR/Cas9 screening will help reveal cellular response to drugs.

## Figures and Tables

**Table:** 

**CRISPR**	Clustered regularly interspaced short palindromic repeats
**Cas9**	CRISPR associated nuclease 9
**LOF**	Loss-of-function
**GOF**	Gain-of-function
**sgRNA**	Single-guide RNA
**DSBs**	DNA double-strand break
**CRISPRa**	CRISPR activation
**CRISPRi**	CRISPR inhibition
**GECKO**	Genome-scale CRISPR Knock-Out
**SAM**	Synergistic activation mediator
**MAPK**	Mitogen-Activated Protein Kinase
**PARPi**	Poly (ADP-ribose) polymerase inhibitors
**ICIs**	Immune checkpoint inhibitors
**CDKI**	Cyclin-dependent kinase inhibitors
**RKT**	Receptor tyrosine kinase
**RAS**	Rat sarcoma
**RAF**	Rapidly Accelerated Fibrosarcoma
**MEK**	Mitogen-activated protein kinase
**ERK**	Extracellular-signal regulated protein kinase
**BRAF**	B-Raf proto-oncogene
**EGFR**	Epidermal growth factor receptor
**VEGFR**	Vascular endothelial growth factor receptor
**FGFR**	Fibroblast Growth Factor Receptor
**MET**	MET proto-oncogene
**KIT**	KIT proto-oncogene
**TKI**	Tyrosine kinase inhibitor
**SHOC2**	SHOC2 leucine-rich repeat scaffold protein
**MRAS**	Muscle RAS oncogene
**PP1c**	Protein phosphatase 1
**SCRIB**	Scribble planar cell polarity protein
**NSCLC**	Non-Small Cell Lung Carcinoma
**RIC8A**	RIC8 guanine nucleotide exchange factor A
**ARIH2**	Ariadne RBR E3 ubiquitin protein ligase 2
**EMT**	Epithelial to mesenchymal transition
**NF1**	Neurofibromin 1
**DUSP9**	Dual specificity phosphatase 9
**HCC**	Hepatocellular carcinoma
**PI3K**	Phosphoinositide 3-Kinase
**AKT**	Protein Kinase B
**DUSP4**	Dual specificity phosphatase 4
**PTEN**	Phosphatase and tensin homolog
**FTase**	Farnesyltransferase
**ROS**	Reactive oxygen species
**KEAP1**	Kelch-like ECH associated protein 1
**Nrf2**	Nuclear factor erythroid 2-related factor 2
**FGF21**	Fibroblast growth factor 21
**ALK**	Anaplastic lymphoma kinase
**MTX1**	Metaxin 1
**CDC37L1**	Cell division cycle 37 like 1
**PPIA**	Peptidylprolyl isomerase A
**SGOL1**	Shugoshin 1
**PHGDH**	Phosphoglycerate dehydrogenase
**SSP**	Serine synthesis pathway
**LRP8**	LDL receptor-related protein
**CIC**	Capicua transcriptional repressor
**FBXO42**	F-box protein 42
**GRB7**	Growth factor receptor bound protein 7
**PLK1**	Polo-like kinase 1
**PARP1**	[oly (ADP-ribose) polymerase 1
**TIGAR**	TP53-induced glycolysis regulatory phosphatase
**ALC1**	Amplification in liver cancer1
**ARH3**	ADP-ribosylserine hydrolase
**PSMA6**	Proteasome 20S subunit alpha 6
**SH3D21**	SH3 domain containing 21
**DCK**	Deoxycytidine kinase
**MTA3**	The metastasis associated 1 family member 3
**CRIP2**	Cysteine-rich protein 2
**ELP5**	Elongator acetyltransferase complex subunit 5
**HnRNPQ**	Heterogeneous nuclear ribonucleoprotein Q
**HDAC1**	Histone deacetylase 1
**ABCG2**	ATP binding cassette subfamily G member 2
**MSH2**	MutS homolog 2
**ARIH1**	Ariadne1 homolog
**SULF1**	Sulfatase 1
**TMZ**	Temozolomide
**E2F6**	E2F transcription factor 6
**PTX**	Paclitaxel
**TCEAL1**	Transcription elongation factor A-like 1
**SSR3**	Signal sequence receptor subunit 3
**PD-1/PD-L1**	Programmed death protein 1/programmed death ligand 1
**CMTM6**	CKLF like MARVEL transmembrane domain containing 6 protein
**SOCS1**	Suppressor of cytokine signaling 1
**MLLT6**	Myeloid/lymphoid or mixed-lineage leukemia; translocated to, 6
**CDK**	Cell cycle-dependent protein kinases
**MCL1**	Myeloid cell leukemia sequence 1

## References

[B1] Abu Samaan T. M. Samec M. Liskova A. Kubatka P. Büsselberg D. (2019). Paclitaxel’s mechanistic and clinical effects on breast cancer. Biomolecules 9, 789. 10.3390/biom9120789 31783552 PMC6995578

[B2] Akbari Kordkheyli V. Rashidi M. Shokri Y. Fallahpour S. Variji A. Nabipour Ghara E. (2022). CRISPER/CAS system, a novel tool of targeted therapy of drug-resistant lung cancer. Adv. Pharm. Bull. 12, 262–273. 10.34172/apb.2022.027 35620343 PMC9106967

[B3] Bakke J. Wright W. C. Zamora A. E. Oladimeji P. Crawford J. C. Brewer C. T. (2019). Genome-wide CRISPR screen reveals PSMA6 to be an essential gene in pancreatic cancer cells. BMC Cancer 19, 253. 10.1186/s12885-019-5455-1 30898113 PMC6429770

[B4] Barazas M. Annunziato S. Pettitt S. J. de Krijger I. Ghezraoui H. Roobol S. J. (2018). The CST complex mediates end protection at double-strand breaks and promotes PARP inhibitor sensitivity in BRCA1-deficient cells. Cell Rep. 23, 2107–2118. 10.1016/j.celrep.2018.04.046 29768208 PMC5972230

[B5] Bi G. Liang J. Zhao M. Zhang H. Jin X. Lu T. (2022). miR-6077 promotes cisplatin/pemetrexed resistance in lung adenocarcinoma via CDKN1A/cell cycle arrest and KEAP1/ferroptosis pathways. Mol. Ther. Nucleic Acids 28, 366–386. 10.1016/j.omtn.2022.03.020 35505963 PMC9035384

[B6] Burr M. L. Sparbier C. E. Chan Y.-C. Williamson J. C. Woods K. Beavis P. A. (2017). CMTM6 maintains the expression of PD-L1 and regulates anti-tumour immunity. Nature 549, 101–105. 10.1038/nature23643 28813417 PMC5706633

[B7] Cai J. Chen J. Wu T. Cheng Z. Tian Y. Pu C. (2020). Genome-scale CRISPR activation screening identifies a role of LRP8 in Sorafenib resistance in Hepatocellular carcinoma. Biochem. Biophys. Res. Commun. 526, 1170–1176. 10.1016/j.bbrc.2020.04.040 32312520

[B8] Cargnello M. Roux P. P. (2011). Activation and function of the MAPKs and their substrates, the MAPK-activated protein kinases. Microbiol. Mol. Biol. Rev. 75, 50–83. 10.1128/MMBR.00031-10 21372320 PMC3063353

[B9] Chen J. Jiang S. Shao H. Li B. Ji T. Staiculescu D. (2022). CRISPR-Cas9-based genome-wide screening identified novel targets for treating sorafenib-resistant hepatocellular carcinoma: a cross-talk between FGF21 and the NRF2 pathway. Sci. China Life Sci. 65, 1998–2016. 10.1007/s11427-021-2067-7 35380342

[B10] Daggubati V. Hochstelter J. Bommireddy A. Choudhury A. Krup A. L. Kaur P. (2021). Smoothened-activating lipids drive resistance to CDK4/6 inhibition in Hedgehog-associated medulloblastoma cells and preclinical models. J. Clin. Invest. 131, 141171. 10.1172/JCI141171 33476305 PMC7954583

[B11] Datlinger P. Rendeiro A. F. Schmidl C. Krausgruber T. Traxler P. Klughammer J. (2017). Pooled CRISPR screening with single-cell transcriptome readout. Nat. Methods 14, 297–301. 10.1038/nmeth.4177 28099430 PMC5334791

[B12] Dev H. Chiang T.-W. W. Lescale C. de Krijger I. Martin A. G. Pilger D. (2018). Shieldin complex promotes DNA end-joining and counters homologous recombination in BRCA1-null cells. Nat. Cell Biol. 20, 954–965. 10.1038/s41556-018-0140-1 30022119 PMC6145444

[B13] Dmello C. Sonabend A. Arrieta V. A. Zhang D. Y. Kanojia D. Chen L. (2022). Translocon-associated protein subunit SSR3 determines and predicts susceptibility to paclitaxel in breast cancer and glioblastoma. Clin. Cancer Res. 28, 3156–3169. 10.1158/1078-0432.CCR-21-2563 35552677 PMC10191221

[B14] Doench J. G. (2018). Am I ready for CRISPR? A user’s guide to genetic screens. Nat. Rev. Genet. 19, 67–80. 10.1038/nrg.2017.97 29199283

[B15] Dong M. B. Wang G. Chow R. D. Ye L. Zhu L. Dai X. (2019). Systematic immunotherapy target discovery using genome-scale *in vivo* CRISPR screens in CD8 T cells. Cell 178, 1189–1204. 10.1016/j.cell.2019.07.044 31442407 PMC6719679

[B16] Fang P. De Souza C. Minn K. Chien J. (2019). Genome-scale CRISPR knockout screen identifies TIGAR as a modifier of PARP inhibitor sensitivity. Commun. Biol. 2, 335. 10.1038/s42003-019-0580-6 31508509 PMC6733792

[B17] Gautron A. Bachelot L. Aubry M. Leclerc D. Quéméner A. M. Corre S. (2021). CRISPR screens identify tumor-promoting genes conferring melanoma cell plasticity and resistance. EMBO Mol. Med. 13, e13466. 10.15252/emmm.202013466 33724679 PMC8103100

[B18] Georgiou A. Stewart A. Cunningham D. Banerji U. Whittaker S. R. (2020). Inactivation of NF1 promotes resistance to EGFR inhibition in KRAS/NRAS/BRAFV600 -Wild-Type colorectal cancer. Mol. Cancer Res. 18, 835–846. 10.1158/1541-7786.MCR-19-1201 32098826 PMC7611272

[B19] Goh C. J. H. Wong J. H. El Farran C. Tan B. X. Coffill C. R. Loh Y.-H. (2021). Identification of pathways modulating vemurafenib resistance in melanoma cells via a genome-wide CRISPR/Cas9 screen. G3 (Bethesda) 11, jkaa069. 10.1093/g3journal/jkaa069 33604667 PMC8022920

[B20] Goodspeed A. Jean A. Costello J. C. (2019). A whole-genome CRISPR screen identifies a role of MSH2 in cisplatin-mediated cell death in muscle-invasive bladder cancer. Eur. Urol. 75, 242–250. 10.1016/j.eururo.2018.10.040 30414698 PMC6339584

[B21] Gottesman M. M. (2002). Mechanisms of cancer drug resistance. Annu. Rev. Med. 53, 615–627. 10.1146/annurev.med.53.082901.103929 11818492

[B22] Gu S. S. Zhang W. Wang X. Jiang P. Traugh N. Li Z. (2021). Therapeutically increasing MHC-I expression potentiates immune checkpoint blockade. Cancer Discov. 11, 1524–1541. 10.1158/2159-8290.CD-20-0812 33589424 PMC8543117

[B23] Han X. Liu Z. Zhao L. Wang F. Yu Y. Yang J. (2016). Microfluidic cell deformability assay for rapid and efficient kinase screening with the CRISPR-cas9 system. Angew. Chem. 128, 8703–8707. 10.1002/ange.201601984 PMC4945455 27258939

[B24] Hayes T. K. Luo F. Cohen O. Goodale A. B. Lee Y. Pantel S. (2019). A functional landscape of resistance to MEK1/2 and CDK4/6 inhibition in NRAS-mutant melanoma. Cancer Res. 79, 2352–2366. 10.1158/0008-5472.CAN-18-2711 30819666 PMC7227487

[B25] Hsu P. D. Lander E. S. Zhang F. (2014). Development and applications of CRISPR-cas9 for genome engineering. Cell 157, 1262–1278. 10.1016/j.cell.2014.05.010 24906146 PMC4343198

[B26] Huang K. Liu X. Li Y. Wang Q. Zhou J. Wang Y. (2019). Genome-wide CRISPR-cas9 screening identifies NF-κB/E2F6 responsible for EGFRvIII-associated temozolomide resistance in glioblastoma. Adv. Sci. (Weinh) 6, 1900782. 10.1002/advs.201900782 31508283 PMC6724471

[B27] Huang S. Ma Z. Zhou Q. Wang A. Gong Y. Li Z. (2022). Genome-wide CRISPR/Cas9 library screening identified that DUSP4 deficiency induces lenvatinib resistance in hepatocellular carcinoma. Int. J. Biol. Sci. 18, 4357–4371. 10.7150/ijbs.69969 35864956 PMC9295068

[B28] Ipsen M. B. Sørensen E. M. G. Thomsen E. A. Weiss S. Haldrup J. Dalby A. (2022). A genome-wide CRISPR-Cas9 knockout screen identifies novel PARP inhibitor resistance genes in prostate cancer. Oncogene 41, 4271–4281. 10.1038/s41388-022-02427-2 35933519

[B29] Jenkins R. W. Barbie D. A. Flaherty K. T. (2018). Mechanisms of resistance to immune checkpoint inhibitors. Br. J. Cancer 118, 9–16. 10.1038/bjc.2017.434 29319049 PMC5765236

[B30] Joung J. Engreitz J. M. Konermann S. Abudayyeh O. O. Verdine V. K. Aguet F. (2017a). Genome-scale activation screen identifies a lncRNA locus regulating a gene neighbourhood. Nature 548, 343–346. 10.1038/nature23451 28792927 PMC5706657

[B31] Joung J. Konermann S. Gootenberg J. S. Abudayyeh O. O. Platt R. J. Brigham M. D. (2017b). Genome-scale CRISPR-Cas9 knockout and transcriptional activation screening. Nat. Protoc. 12, 828–863. 10.1038/nprot.2017.016 28333914 PMC5526071

[B32] Juhász S. Smith R. Schauer T. Spekhardt D. Mamar H. Zentout S. (2020). The chromatin remodeler ALC1 underlies resistance to PARP inhibitor treatment. Sci. Adv. 6, eabb8626. 10.1126/sciadv.abb8626 33355125 PMC11206534

[B33] Kabir S. Cidado J. Andersen C. Dick C. Lin P.-C. Mitros T. (2019). The CUL5 ubiquitin ligase complex mediates resistance to CDK9 and MCL1 inhibitors in lung cancer cells. Elife 8, e44288. 10.7554/eLife.44288 31294695 PMC6701926

[B34] Ko T. Li S. (2019). Genome-wide screening identifies novel genes and biological processes implicated in cisplatin resistance. FASEB J. 33, 7143–7154. 10.1096/fj.201801534RR 30844312

[B35] Konermann S. Brigham M. D. Trevino A. E. Joung J. Abudayyeh O. O. Barcena C. (2015). Genome-scale transcriptional activation by an engineered CRISPR-Cas9 complex. Nature 517, 583–588. 10.1038/nature14136 25494202 PMC4420636

[B36] Krall E. B. Wang B. Munoz D. M. Ilic N. Raghavan S. Niederst M. J. (2017). KEAP1 loss modulates sensitivity to kinase targeted therapy in lung cancer. Elife 6, e18970. 10.7554/eLife.18970 28145866 PMC5305212

[B37] Kriegsman B. A. Vangala P. Chen B. J. Meraner P. Brass A. L. Garber M. (2019). Frequent loss of IRF2 in cancers leads to immune evasion through decreased MHC class I antigen presentation and increased PD-L1 expression. J. Immunol. 203, 1999–2010. 10.4049/jimmunol.1900475 31471524 PMC6761035

[B38] Kwon J. J. Hajian B. Bian Y. Young L. C. Amor A. J. Fuller J. R. (2022). Structure-function analysis of the SHOC2-MRAS-PP1C holophosphatase complex. Nature 609, 408–415. 10.1038/s41586-022-04928-2 35831509 PMC9694338

[B39] Lan H. Liu Y. Liu J. Wang X. Guan Z. Du J. (2021). Tumor-associated macrophages promote oxaliplatin resistance via METTL3-mediated m6A of TRAF5 and necroptosis in colorectal cancer. Mol. Pharm. 18, 1026–1037. 10.1021/acs.molpharmaceut.0c00961 33555197

[B40] Lee J. Choi A. Cho S.-Y. Jun Y. Na D. Lee A. (2021b). Genome-scale CRISPR screening identifies cell cycle and protein ubiquitination processes as druggable targets for erlotinib-resistant lung cancer. Mol. Oncol. 15, 487–502. 10.1002/1878-0261.12853 33188726 PMC7858278

[B41] Lee J. B. Ha S.-J. Kim H. R. (2021a). Clinical insights into novel immune checkpoint inhibitors. Front. Pharmacol. 12, 681320. 10.3389/fphar.2021.681320 34025438 PMC8139127

[B42] Lee S. Y. (2016). Temozolomide resistance in glioblastoma multiforme. Genes Dis. 3, 198–210. 10.1016/j.gendis.2016.04.007 30258889 PMC6150109

[B43] Li L. Yu S. Chen J. Quan M. Gao Y. Li Y. (2022). miR-15a and miR-20b sensitize hepatocellular carcinoma cells to sorafenib through repressing CDC37L1 and consequent PPIA downregulation. Cell Death Discov. 8, 297. 10.1038/s41420-022-01094-2 35760798 PMC9237098

[B44] Li L. Yu S. Hu Q. Hai Y. Li Y. (2021). Genome-scale CRISPRa screening identifies MTX1 as a contributor for sorafenib resistance in hepatocellular carcinoma by augmenting autophagy. Int. J. Biol. Sci. 17, 3133–3144. 10.7150/ijbs.62393 34421355 PMC8375235

[B45] Li W. Xu H. Xiao T. Cong L. Love M. I. Zhang F. (2014). MAGeCK enables robust identification of essential genes from genome-scale CRISPR/Cas9 knockout screens. Genome Biol. 15, 554. 10.1186/s13059-014-0554-4 25476604 PMC4290824

[B46] Lian B. Pei Y.-C. Jiang Y.-Z. Xue M.-Z. Li D.-Q. Li X.-G. (2020). Truncated HDAC9 identified by integrated genome-wide screen as the key modulator for paclitaxel resistance in triple-negative breast cancer. Theranostics 10, 11092–11109. 10.7150/thno.44997 33042272 PMC7532680

[B47] Ling A. Gruener R. F. Fessler J. Huang R. S. (2018). More than fishing for a cure: the promises and pitfalls of high throughput cancer cell line screens. Pharmacol. Ther. 191, 178–189. 10.1016/j.pharmthera.2018.06.014 29953899 PMC7001883

[B48] Lu Y. Shen H. Huang W. He S. Chen J. Zhang D. (2021). Genome-scale CRISPR-Cas9 knockout screening in hepatocellular carcinoma with lenvatinib resistance. Cell Death Discov. 7, 359. 10.1038/s41420-021-00747-y 34795217 PMC8602346

[B49] MacLeod G. Bozek D. A. Rajakulendran N. Monteiro V. Ahmadi M. Steinhart Z. (2019). Genome-wide CRISPR-cas9 screens expose genetic vulnerabilities and mechanisms of temozolomide sensitivity in glioblastoma stem cells. Cell Rep. 27, 971–986. 10.1016/j.celrep.2019.03.047 30995489

[B50] Makhov P. Sohn J. A. Serebriiskii I. G. Fazliyeva R. Khazak V. Boumber Y. (2020). CRISPR/Cas9 genome-wide loss-of-function screening identifies druggable cellular factors involved in sunitinib resistance in renal cell carcinoma. Br. J. Cancer 123, 1749–1756. 10.1038/s41416-020-01087-x 32968206 PMC7723036

[B51] Manguso R. T. Pope H. W. Zimmer M. D. Brown F. D. Yates K. B. Miller B. C. (2017). *In vivo* CRISPR screening identifies Ptpn2 as a cancer immunotherapy target. Nature 547, 413–418. 10.1038/nature23270 28723893 PMC5924693

[B52] Masoudi M. Seki M. Yazdanparast R. Yachie N. Aburatani H. (2019). A genome-scale CRISPR/Cas9 knockout screening reveals SH3D21 as a sensitizer for gemcitabine. Sci. Rep. 9, 19188. 10.1038/s41598-019-55893-2 31844142 PMC6915784

[B53] Mayayo-Peralta I. Faggion B. Hoekman L. Morris B. Lieftink C. Goldsbrough I. (2021). Ribociclib induces broad chemotherapy resistance and EGFR dependency in ESR1 wildtype and mutant breast cancer. Cancers (Basel) 13, 6314. 10.3390/cancers13246314 34944934 PMC8699146

[B54] Mini E. Nobili S. Caciagli B. Landini I. Mazzei T. (2006). Cellular pharmacology of gemcitabine. Ann. Oncol. 17 (Suppl. 5), v7–v12. 10.1093/annonc/mdj941 16807468

[B55] Mojica F. J. M. Montoliu L. (2016). On the origin of CRISPR-cas technology: from prokaryotes to mammals. Trends Microbiol. 24, 811–820. 10.1016/j.tim.2016.06.005 27401123

[B56] Munoz D. M. Cassiani P. J. Li L. Billy E. Korn J. M. Jones M. D. (2016). CRISPR screens provide a comprehensive assessment of cancer vulnerabilities but generate false-positive hits for highly amplified genomic regions. Cancer Discov. 6, 900–913. 10.1158/2159-8290.CD-16-0178 27260157

[B57] Nagler A. Vredevoogd D. W. Alon M. Cheng P. F. Trabish S. Kalaora S. (2020). A genome-wide CRISPR screen identifies FBXO42 involvement in resistance toward MEK inhibition in NRAS-mutant melanoma. Pigment. Cell Melanoma Res. 33, 334–344. 10.1111/pcmr.12825 31549767 PMC7383499

[B58] Ning G. Zhu Q. Kang W. Lee H. Maher L. Suh Y.-S. (2021). A novel treatment strategy for lapatinib resistance in a subset of HER2-amplified gastric cancer. BMC Cancer 21, 923. 10.1186/s12885-021-08283-9 34399705 PMC8366014

[B59] Noordermeer S. M. van Attikum H. (2019). PARP inhibitor resistance: a tug-of-war in BRCA-mutated cells. Trends Cell Biol. 29, 820–834. 10.1016/j.tcb.2019.07.008 31421928

[B60] Okada M. Chikuma S. Kondo T. Hibino S. Machiyama H. Yokosuka T. (2017). Blockage of core fucosylation reduces cell-surface expression of PD-1 and promotes anti-tumor immune responses of T cells. Cell Rep. 20, 1017–1028. 10.1016/j.celrep.2017.07.027 28768188

[B61] Ouyang Q. Liu Y. Tan J. Li J. Yang D. Zeng F. (2019). Loss of ZNF587B and SULF1 contributed to cisplatin resistance in ovarian cancer cell lines based on Genome-scale CRISPR/Cas9 screening. Am. J. Cancer Res. 9, 988–998. 31218106 PMC6556596

[B62] Pettitt S. J. Krastev D. B. Brandsma I. Dréan A. Song F. Aleksandrov R. (2018). Genome-wide and high-density CRISPR-Cas9 screens identify point mutations in PARP1 causing PARP inhibitor resistance. Nat. Commun. 9, 1849. 10.1038/s41467-018-03917-2 29748565 PMC5945626

[B63] Ramaker R. C. Hardigan A. A. Gordon E. R. Wright C. A. Myers R. M. Cooper S. J. (2021). Pooled CRISPR screening in pancreatic cancer cells implicates co-repressor complexes as a cause of multiple drug resistance via regulation of epithelial-to-mesenchymal transition. BMC Cancer 21, 632. 10.1186/s12885-021-08388-1 34049503 PMC8164247

[B64] Raoof S. Mulford I. J. Frisco-Cabanos H. Nangia V. Timonina D. Labrot E. (2019). Targeting FGFR overcomes EMT-mediated resistance in EGFR mutant non-small cell lung cancer. Oncogene 38, 6399–6413. 10.1038/s41388-019-0887-2 31324888 PMC6742540

[B65] Regad T. (2015). Targeting RTK signaling pathways in cancer. Cancers 7, 1758–1784. 10.3390/cancers7030860 26404379 PMC4586793

[B66] Rocha C. R. R. Reily Rocha A. Molina Silva M. Rodrigues Gomes L. Teatin Latancia M. Andrade Tomaz M. (2020). Revealing temozolomide resistance mechanisms via genome-wide CRISPR libraries. Cells 9, E2573. 10.3390/cells9122573 PMC7760831 33271924

[B67] Rushworth L. K. Harle V. Repiscak P. Clark W. Shaw R. Hall H. (2020). *In vivo* CRISPR/Cas9 knockout screen: TCEAL1 silencing enhances docetaxel efficacy in prostate cancer. Life Sci. Alliance 3, e202000770. 10.26508/lsa.202000770 33033111 PMC7556750

[B68] Sanjana N. E. Shalem O. Zhang F. (2014). Improved vectors and genome-wide libraries for CRISPR screening. Nat. Methods 11, 783–784. 10.1038/nmeth.3047 25075903 PMC4486245

[B69] Sarr A. Bré J. Um I. H. Chan T. H. Mullen P. Harrison D. J. (2019). Genome-scale CRISPR/Cas9 screen determines factors modulating sensitivity to ProTide NUC-1031. Sci. Rep. 9, 7643. 10.1038/s41598-019-44089-3 31113993 PMC6529431

[B70] Shalem O. Sanjana N. E. Zhang F. (2015). High-throughput functional genomics using CRISPR–Cas9. Nat. Rev. Genet. 16, 299–311. 10.1038/nrg3899 25854182 PMC4503232

[B71] Sharma S. Petsalaki E. (2018). Application of CRISPR-cas9 based genome-wide screening approaches to study cellular signalling mechanisms. Int. J. Mol. Sci. 19, 933. 10.3390/ijms19040933 29561791 PMC5979383

[B72] Shi Z.-D. Hao L. Han X.-X. Wu Z.-X. Pang K. Dong Y. (2022). Targeting HNRNPU to overcome cisplatin resistance in bladder cancer. Mol. Cancer 21, 37. 10.1186/s12943-022-01517-9 35130920 PMC8819945

[B73] Skripova V. Vlasenkova R. Zhou Y. Astsaturov I. Kiyamova R. (2022). Identification of new regulators of pancreatic cancer cell sensitivity to oxaliplatin and cisplatin. Molecules 27, 1289. 10.3390/molecules27041289 35209078 PMC8875979

[B74] Sofer S. Lamkiewicz K. Armoza Eilat S. Partouche S. Marz M. Moskovits N. (2022). A genome-wide CRISPR activation screen reveals Hexokinase 1 as a critical factor in promoting resistance to multi-kinase inhibitors in hepatocellular carcinoma cells. FASEB J. 36, e22191. 10.1096/fj.202101507RR 35147243

[B75] Sreevalsan S. Döring M. Paszkowski-Rogacz M. Brux M. Blanck C. Meyer M. (2020). MLLT6 maintains PD-L1 expression and mediates tumor immune resistance. EMBO Rep. 21, e50155. 10.15252/embr.202050155 33063451 PMC7726806

[B76] Stover E. H. Baco M. B. Cohen O. Li Y. Y. Christie E. L. Bagul M. (2019). Pooled genomic screens identify anti-apoptotic genes as targetable mediators of chemotherapy resistance in ovarian cancer. Mol. Cancer Res. 17, 2281–2293. 10.1158/1541-7786.MCR-18-1243 31462500 PMC6825578

[B77] Sun W. He B. Yang B. Hu W. Cheng S. Xiao H. (2018). Genome-wide CRISPR screen reveals SGOL1 as a druggable target of sorafenib-treated hepatocellular carcinoma. Lab. Invest. 98, 734–744. 10.1038/s41374-018-0027-6 29467456

[B78] Suresh S. Chen B. Zhu J. Golden R. J. Lu C. Evers B. M. (2020). eIF5B drives integrated stress response-dependent translation of PD-L1 in lung cancer. Nat. Cancer 1, 533–545. 10.1038/s43018-020-0056-0 32984844 PMC7511089

[B79] Šuštić T. van Wageningen S. Bosdriesz E. Reid R. J. D. Dittmar J. Lieftink C. (2018). A role for the unfolded protein response stress sensor ERN1 in regulating the response to MEK inhibitors in KRAS mutant colon cancers. Genome Med. 10, 90. 10.1186/s13073-018-0600-z 30482246 PMC6258447

[B80] Terai H. Hamamoto J. Emoto K. Masuda T. Manabe T. Kuronuma S. (2021). SHOC2 is a critical modulator of sensitivity to EGFR-TKIs in non-small cell lung cancer cells. Mol. Cancer Res. 19, 317–328. 10.1158/1541-7786.MCR-20-0664 33106373

[B81] Tong Z. Sathe A. Ebner B. Qi P. Veltkamp C. Gschwend J. E. (2019). Functional genomics identifies predictive markers and clinically actionable resistance mechanisms to CDK4/6 inhibition in bladder cancer. J. Exp. Clin. Cancer Res. 38, 322. 10.1186/s13046-019-1322-9 31331377 PMC6647307

[B82] Wan C. Mahara S. Sun C. Doan A. Chua H. K. Xu D. (2021). Genome-scale CRISPR-Cas9 screen of Wnt/β-catenin signaling identifies therapeutic targets for colorectal cancer. Sci. Adv. 7, eabf2567. 10.1126/sciadv.abf2567 34138730 PMC8133758

[B83] Wang B. Krall E. B. Aguirre A. J. Kim M. Widlund H. R. Doshi M. B. (2017). ATXN1L, CIC, and ETS transcription factors modulate sensitivity to MAPK pathway inhibition. Cell Rep. 18, 1543–1557. 10.1016/j.celrep.2017.01.031 28178529 PMC5313047

[B84] Wang L. Zhao X. Fu J. Xu W. Yuan J. (2021a). The role of tumour metabolism in cisplatin resistance. Front. Mol. Biosci. 8, 691795. 10.3389/fmolb.2021.691795 34250022 PMC8261055

[B85] Wang N. Ma T. Yu B. (2023). Targeting epigenetic regulators to overcome drug resistance in cancers. Sig Transduct. Target Ther. 8, 69–24. 10.1038/s41392-023-01341-7 PMC9935618 36797239

[B86] Wang W. Wang X. (2017). Single-cell CRISPR screening in drug resistance. Cell Biol. Toxicol. 33, 207–210. 10.1007/s10565-017-9396-7 28474250

[B87] Wang Y. Wang F. Wang L. Qiu S. Yao Y. Yan C. (2021b). NAD+ supplement potentiates tumor-killing function by rescuing defective TUB-mediated NAMPT transcription in tumor-infiltrated T cells. Cell Rep. 36, 109516. 10.1016/j.celrep.2021.109516 34380043

[B88] Ward R. A. Fawell S. Floc’h N. Flemington V. McKerrecher D. Smith P. D. (2021). Challenges and opportunities in cancer drug resistance. Chem. Rev. 121, 3297–3351. 10.1021/acs.chemrev.0c00383 32692162

[B89] Wei L. Lee D. Law C.-T. Zhang M. S. Shen J. Chin D. W.-C. (2019). Genome-wide CRISPR/Cas9 library screening identified PHGDH as a critical driver for Sorafenib resistance in HCC. Nat. Commun. 10, 4681. 10.1038/s41467-019-12606-7 31615983 PMC6794322

[B90] Wroblewska A. Dhainaut M. Ben-Zvi B. Rose S. A. Park E. S. Amir E.-A. D. (2018). Protein barcodes enable high-dimensional single-cell CRISPR screens. Cell 175, 1141–1155. 10.1016/j.cell.2018.09.022 30343902 PMC6319269

[B91] Wu L. Ge Y. Yuan Y. Li H. Sun H. Xu C. (2022). Genome-wide CRISPR screen identifies MTA3 as an inducer of gemcitabine resistance in pancreatic ductal adenocarcinoma. Cancer Lett. 548, 215864. 10.1016/j.canlet.2022.215864 35981571

[B92] Xu S. Zhan M. Jiang C. He M. Yang L. Shen H. (2019). Genome-wide CRISPR screen identifies ELP5 as a determinant of gemcitabine sensitivity in gallbladder cancer. Nat. Commun. 10, 5492. 10.1038/s41467-019-13420-x 31792210 PMC6889377

[B93] Yang H. Liu B. Liu D. Yang Z. Zhang S. Xu P. (2022). Genome-wide CRISPR screening identifies DCK and CCNL1 as genes that contribute to gemcitabine resistance in pancreatic cancer. Cancers (Basel) 14, 3152. 10.3390/cancers14133152 35804923 PMC9264918

[B94] Yu C. Luo D. Yu J. Zhang M. Zheng X. Xu G. (2022). Genome-wide CRISPR-cas9 knockout screening identifies GRB7 as a driver for MEK inhibitor resistance in KRAS mutant colon cancer. Oncogene 41, 191–203. 10.1038/s41388-021-02077-w 34718347 PMC8732282

[B95] Yu J. S. L. Yusa K. (2019). Genome-wide CRISPR-Cas9 screening in mammalian cells. Methods 164–165, 29–35. 10.1016/j.ymeth.2019.04.015 31034882

[B96] Zeng H. Castillo-Cabrera J. Manser M. Lu B. Yang Z. Strande V. (2019). Genome-wide CRISPR screening reveals genetic modifiers of mutant EGFR dependence in human NSCLC. Elife 8, e50223. 10.7554/eLife.50223 31741433 PMC6927754

[B97] Zhai B. Sun X.-Y. (2013). Mechanisms of resistance to sorafenib and the corresponding strategies in hepatocellular carcinoma. World J. Hepatol. 5, 345–352. 10.4254/wjh.v5.i7.345 23898367 PMC3724962

[B98] Zhang J.-P. Song Z. Wang H.-B. Lang L. Yang Y.-Z. Xiao W. (2019). A novel model of controlling PD-L1 expression in ALK+ anaplastic large cell lymphoma revealed by CRISPR screening. Blood 134, 171–185. 10.1182/blood.2019001043 31151983 PMC6624970

[B99] Zhang Q. Liu H. (2020). Functioning mechanisms of Shugoshin-1 in centromeric cohesion during mitosis. Essays Biochem. 64, 289–297. 10.1042/EBC20190077 32451529 PMC7483588

[B100] Zheng A. Chevalier N. Calderoni M. Dubuis G. Dormond O. Ziros P. G. (2019). CRISPR/Cas9 genome-wide screening identifies KEAP1 as a sorafenib, lenvatinib, and regorafenib sensitivity gene in hepatocellular carcinoma. Oncotarget 10, 7058–7070. 10.18632/oncotarget.27361 31903165 PMC6925031

[B101] Zhong Z. Harmston N. Wood K. C. Madan B. Virshup D. M. (2022). A p300/GATA6 axis determines differentiation and Wnt dependency in pancreatic cancer models. J. Clin. Invest. 132, e156305. 10.1172/JCI156305 35536676 PMC9197518

[B102] Zimmermann M. Murina O. Reijns M. A. M. Agathanggelou A. Challis R. Tarnauskaitė Ž. (2018). CRISPR screens identify genomic ribonucleotides as a source of PARP-trapping lesions. Nature 559, 285–289. 10.1038/s41586-018-0291-z 29973717 PMC6071917

