# Supplementary material for: Genome-wide CRISPR/Cas9 screening for drug resistance in tumors
Source: Front Pharmacol. 2023 Nov 21;14:1284610. doi: 10.3389/fphar.2023.1284610 (PMC10710899; doi:10.3389/fphar.2023.1284610)
Supplement: Supplementary file 1 [file Table1.docx]

| Supplementary Material  Supplementary Table 1. Summary of CRISPR screen with RTK inhibitors | | | | | | | | |
| --- | --- | --- | --- | --- | --- | --- | --- | --- |
| Cancer | | **Gene** | **CRISPR** | **Cell line** | **Drug** | **Related pathway** | **Effect** | **Reference** |
| NSCLC | SHOC2 | | KNOCKOUT | PC9 | (TKI)Erlotinib | PP1, MRAS, and SCRIB pathway | Knockout of SHOC2 sensitized the cells | (19) |
| NSCLC | RIC8A/ARIH2 | | KNOCKOUT | HCC827 | (TKI)Erlotinib | YAP, PI3K-mTOR and RAS-MAPK signaling pathways | Knockout of RIC8A sensitized cells/knockout of ARIH2 induced resistance | (21) |
| CRC | NF1 | | KNOCKOUT | DIFI | (TKI)Gefitinib | MAPK and PI3K pathway | Reduced NF1 expression sustained signaling through the MAPK pathway | (22) |
| NSCLC | MDM4/PSMA6/PSMB6/ANAPC5/CDK1 | | KNOCKOUT | NCI-H820 | (TKI)Erlotinib | Protein ubiquitination and proteasomal pathway &cell cycle processes | Knockout of MDM4&PSMA6 sensitized the cells | (23) |
| NSCLC | FGFR1 | | KNOCKOUT | Mesenchymal cell lines | (TKI)EGF816 | FGFR signaling pathway | Inhibition of FGFR sensitized cells | (24) |

| Supplementary Table 2. Summary of CRISPR screen with multi-targeted RTK inhibitors | | | | | | | |
| --- | --- | --- | --- | --- | --- | --- | --- |
| Cancer | **Gene** | **CRISPR** | **Cell line** | **Drug** | **Related pathway** | **Effect** | **Reference** |
| HCC | NF1&DUSP9 | KNOCKOUT | Huh7 | (TKI)Lenvatinib | PI3K/AKT and MAPK/ERK signaling pathway | Knockout of NF1 and DUSP9 induced resistance | (25) |
| HCC | DUSP4 | KNOCKOUT | HepG2 | (TKI)Lenvatinib | MAPK/ERK pathway | Knockout of DUSP4 Induced resistance | (26) |
| GC | CSK&PTEN | KNOCKOUT | N87/OE19 | (TKI)Lapatinib | PI3K and MAPK pathway | Loss of function mutation of CSK and PTEN re-activate PI3K and MAPK pathways | (27) |
| ccRCC | farnesyltransferase | KNOCKOUT | 786O | (TKI)Sunitinib | / | Knockout of FNTA and FNTB induced resistance | (28) |
| HCC | KEAP1 | KNOCKOUT | Huh7 | (TKI)Sorafenib | KEAP1/Nrf2 pathway | Knockout of KEAP1 induced resistance | (29) |
| HCC | KEAP1/FGF21 | KNOCKOUT | HepG2 | (TKI)Sorafenib | KEAP1/Nrf2/FGF21 pathway | Knockout of KEAP1 induced resistance | (30) |
| LUAD/HCC | KEAP1 | KNOCKOUT | NCI-H1299/CALU1/HCC364/HCC827/MGH-065 | (TKI)Vemurafenib/Trametinib/Erlotinib/ Crizotinib/LDK-378 | KEAP1/Nrf2 pathway | Knockout of KEAP1 induced resistance | (31) |
| LUAD | miR-6077 | KNOCKOUT | A549 | Cisplatin/Pemetrexed | CDKN1A/cell cycle arrest and KEAP1/ferroptosis pathways | MiR-6077 desensitized both A549 and H358 cell lines to CDDP/PEM treatment | (32) |
| HCC | MTX1 | OVEREXPRESSION | HCC-LM3 | (TKI)Sorafenib | MTX1-CISD1 pathway | Overexpression of MTX1 induced resistance | (33) |
| HCC | SGOL1 | KNOCKOUT | Huh7 | (TKI)Sorafenib | Sister chromatid cohesion | Knockout of SGOL1 induced resistance | (34) |
| HCC | PHGDH | KNOCKOUT | MHCC97L | (TKI)Sorafenib | Serine synthesis pathway | Knockout of PHGDH sensitized the cells | (36) |
| HCC | HK1/ITGB5 | OVEREXPRESSION | Huh7 | (TKI)regorafenib | Glycolysis | Overexpression of HK1/ITGB5 induced resistance | (37) |
| HCC | LRP8 | OVEREXPRESSION | Huh7 | (TKI)Sorafenib | ApoE-LRP8 pathway | Overexpression of LRP8 induced resistance | (38) |

| Supplementary Table 3. Summary of CRISPR screen with RAF inhibitors | | | | | | | |
| --- | --- | --- | --- | --- | --- | --- | --- |
| Cancer | **Gene** | **CRISPR** | **Cell line** | **Drug** | **Related pathway** | **Effect** | **Reference** |
| Melanoma | 14 new genes | KNOCKOUT | A375 | Vemurafenib | MAPK pathway | / | (39) |
| Melanoma | EMICERI | OVEREXPRESSION | A375 | Vemurafenib | / | A LncRNA Locus regulates a gene neighborhood | (40) |
| Melanoma | SMAD3, BIRC3, and SLC9A5 | OVEREXPRESSION | 501Mel | Vemurafenib | TGFB signaling pathway | The upregulation of the SMAD3 induced resistance | (41) |

| Supplementary Table 4. Summary of CRISPR screen with MEK1/2 protein inhibitors | | | | | | | |
| --- | --- | --- | --- | --- | --- | --- | --- |
| Cancer | **Gene** | **CRISPR** | **Cell line** | **Drug** | **Related pathway** | **Effect** | **Reference** |
| PDAC | AXN1L/CIC/ETS | KNOCKOUT | PATU8902 and PATU8988T | Trametinib | MAPK pathway inhibition. | Knockout of CIC induced resistance | (42) |
| CRC | GRB7 | KNOCKOUT | HCT116 | AZD6244 | RTK pathway | The combination of PLK1 and MEK inhibitors synergistically inhibited CRC cell proliferation | (45) |

| Supplementary Table 5. Summary of CRISPR screen with PARPi | | | | | | | |
| --- | --- | --- | --- | --- | --- | --- | --- |
| Cancer | **Gene** | **CRISPR** | **Cell line** | **Drug** | **Related pathway** | **Effect** | **Reference** |
| Breast cancer | CTC1/STN1/TEN1 | KNOCKOUT | KB1P-G3/SUM149PT/mES | AZD2461/Olaparib/Talazoparib | DNA double strand break | The CST complex maintains double-strand break end stability and protects telomeric ends. | (47) |
| Breast cancer | C20orf196/ FAM35A | KNOCKOUT | SUM149PT | Olaparib/Alazoparib/AZD2461 | DNA double strand break | The inactivation of two components induced resistance | (48) |
| Ovarian cancer | PARP1 | KNOCKOUT | ES cells | Talazoparib | DNA double strand break | Mutations both within and outside of the PARP1 DNA-binding zinc-finger domains cause PARPi resistance | (49) |
| Cervical adenocarcinoma &Breast cancer | RNASEH2A/RNASEH2B /RNASEH2C | KNOCKOUT | HeLa/ RPE1-hTERT/ SUM149PT | Olaparib | Ribonucleotide excision repair | Knockout of the RNASEH2B sensitized cells | (50) |
| Ovarian cancer | TIGAR | KNOCKOUT | A2780 | Olaparib | TP53 pathway | Knockdown of TIGAR induced sensitivity to Olaparnib | (51) |
| Cervical adenocarcinoma | ALC1 | KNOCKOUT | Hela cells | Olaparib | Single-strand breaks and double-strand breaks | Knockout of ALC1 can sensitize cells | (52) |
| Prostate cancer | PARP1/ARH3/YWHAE/ UBR5 | KNOCKOUT | C4 | Olaparib | Autophagy pathway | Knockdown of PARP1, ARH3, YWHAE induced resistance | (53) |

| Supplementary Table 6. Summary of CRISPR screen with antimetabolites | | | | | | | |
| --- | --- | --- | --- | --- | --- | --- | --- |
| Cancer | **Gene** | **CRISPR** | **Cell line** | **Drug** | **Related pathway** | **Effect** | **Reference** |
| PDAC | PSMA6 | KNOCKOUT | PANC-1 | Gemcitabine | Proteasome pathway | Knockout of PSMA6 sensitized cells | (55) |
| PDAC | SH3D21 | KNOCKOUT | PANC-1 | Gemcitabine | MYC pathway | Knockout of SH3D21 sensitized the cells | (56) |
| PDAC | DCK/CCNL1 | KNOCKOUT | TB32047 | Gemcitabine | ERK/AKT/STAT3 survival pathway | Knockout of CCNL1 induced resistance | (57) |
| PDAC | MTA3 | OVEREXPRESSION | BxPC-3 | Gemcitabine | MTA3-CRIP2-NF-Κb pathway | Overexpression of MTA3 induced resistance | (58) |
| PDAC/ Ovarian cancer | DCK/DCTPP1 | KNOCKOUT | MiaPaCa2 | NUC-1031 | The pyrimidine metabolism pathway | Knockout of DCK Induced resistance | (59) |
| GLBC | ELP5 | KNOCKOUT | NOZ | Gemcitabine | P53 pathway | Knockout of ELP5 Induced resistance | (60) |
| PDAC | HDAC1/ABCG2 | KNOCKOUT &OVEREXPRESSION | Panc-1 and BxPC3 | Gemcitabine, oxaliplatin, irinotecan and 5-fluorouracil | Chromatin remodeling/the regulation of the EMT program | Overexpression of ABCG2 induced resistance | (61) |

| Supplementary Table7. Summary of CRISPR screen with alkylating agents | | | | | | | |
| --- | --- | --- | --- | --- | --- | --- | --- |
| Cancer | **Gene** | **CRISPR** | **Cell line** | **Drug** | **Related pathway** | **Effect** | **Reference** |
| BLCA | MSH2 | KNOCKOUT | MGHU4 | cisplatin | MMR pathway | Knockout of MSH2 induced resistance | (63) |
| BLCA | HNRNPU | KNOCKOUT | T24 | cisplatin | NF1 related signaling pathway | Knockout of HNRNPU sensitized the cells | (64) |
| Melanoma | ZNRF3/ARIH2 | KNOCKOUT | A375 | cisplatin | Wnt β-catenin signaling, | ZNRF3 negatively regulates the Wnt-β catenin pathway | (65) |
| CRC | TRAF5 | KNOCKOUT | HCT-116 | Oxaliplatin | TRAF5-METTL3-m6A and tumor associated macrophages | The m6A of TRAF5 induced resistance | (66) |
| ovary cancer | ZNF587B/SULF1 | KNOCKOUT | SKOV3 and A2780 cells | Cisplatin | / | Knockout of ZNF587B and SULF1 induced resistance | (67) |
| High-grade serous ovarian cancer (HGSOC) | BCL-2 family | KNOCKOUT&OVEREXPRESSION | OVSAHO cells | Cisplatin, paclitaxel | Anti-apoptotic pathway | Overexpression of anti-apoptotic proteins induced resistance | (68) |
| Glioblastoma | NF-κB/E2F6 | KNOCKOUT | U87 | Temozolomide | EGFRvIII/AKT/NF-κB pathway | Overexpression of E2F6 drives a TMZ resistance | (71) |
| Glioblastoma | SOX2/SOX9/DOT1L/SOCS3 | KNOCKOUT | HF7450/HF6562 | Temozolomide | Stemness pathway | / | (72) |
| glioblastoma | MSH2/PTCH2/CLCA2/FZD6/CTNNB1/NRF2 | KNOCKOUT&OVEREXPRESSION | U138MG | Temozolomide | DNA repair/Sonic Hedgehog pathway/Wnt pathway | Overexpression of FZD6, CTNNB1, or NRF2 induced resistance | (73) |

| Supplementary Table 8. Summary of CRISPR screen with mitotic inhibitors | | | | | | | |
| --- | --- | --- | --- | --- | --- | --- | --- |
| Cancer | **Gene** | **CRISPR** | **Cell line** | **Drug** | **Related pathway** | **Effect** | **Reference** |
| Prostate cancer | TCEAL1 | KNOCKOUT | SP1 | Docetaxel | Cell cycle | Knockout of the TECAL1 sensitized cells | (75) |
| Breast cancer | HDAC | KNOCKOUT | MDA-MB-231 | Docetaxel | HDAC-MITR-MEF2A-IL11 pathway | Suppression of HDAC9 augmented paclitaxel-mediated cytotoxic effects | (76) |
| Breast cancer | SSR3 | KNOCKOUT | H4 | Paclitaxel | Translocon-associated Protein Subunit SSR3 | Overexpression of SSR3 sensitized the cells | (77) |

| Supplementary Table 9. Summary of CRISPR screen with CDK inhibitors | | | | | | | | |
| --- | --- | --- | --- | --- | --- | --- | --- | --- |
| Cancer | **Gene** | **CRISPR** | **Cell line** | **Drug** | **Related pathway** | **Effect** | **Reference** | |
| BLCA | / | activation | T24 | Palbociclib | RTK/PI3K-Akt, Ras/MAPK, JAK/STAT/Wnt signaling pathways | / | | (89) |
| NSCLC | CUL5, RNF7 and UBE2F | Knockout | LK2 | AZD5576/AZD5991 | Ubiquitin pathway | The novel role of CRL5 in apoptosis and resistance | | (90) |
| Medulloblastoma | / | Knockout | DAOY | Abemaciclib | Hedgehog pathway | Decreased ribosomal protein expression underlies resistance to CDK6 inhibition | | (91) |
| Melanoma | / | OVEREXPRESSION/Knockout | MELJUSO | Trametinib/Palbociclib | MEK1/2 pathway | The upregulating RTK–RAS–RAF and RTK–PI3K–AKT signaling in NRAS-mutant melanomas | | (92) |
| Breast cancer | / | Knockout | MCF-7 | Ribociclib | EGFR pathway | EGFR activation in ribociclib-induced senescent breast cancer cells | | (93) |
